# ChIP-GSM: Inferring active transcription factor modules to predict functional regulatory elements

**DOI:** 10.1371/journal.pcbi.1009203

**Published:** 2021-07-22

**Authors:** Xi Chen, Andrew F. Neuwald, Leena Hilakivi-Clarke, Robert Clarke, Jianhua Xuan

**Affiliations:** 1 Bradley Department of Electrical and Computer Engineering, Virginia Polytechnic Institute and State University, Arlington, Virginia, United States of America; 2 Center for Computational Biology, Flatiron Institute, Simons Foundation, New York, New York, United States of America; 3 Institute for Genome Sciences and Department Biochemistry & Molecular Biology, University of Maryland School of Medicine, Baltimore, Maryland, United States of America; 4 Hormel Institute, University of Minnesota, Minnesota, United States of America; University of Virginia, UNITED STATES

## Abstract

Transcription factors (TFs) often function as a module including both master factors and mediators binding at cis-regulatory regions to modulate nearby gene transcription. ChIP-seq profiling of multiple TFs makes it feasible to infer functional TF modules. However, when inferring TF modules based on co-localization of ChIP-seq peaks, often many weak binding events are missed, especially for mediators, resulting in incomplete identification of modules. To address this problem, we develop a ChIP-seq data-driven Gibbs Sampler to infer Modules (ChIP-GSM) using a Bayesian framework that integrates ChIP-seq profiles of multiple TFs. ChIP-GSM samples read counts of module TFs iteratively to estimate the binding potential of a module to each region and, across all regions, estimates the module abundance. Using inferred module-region probabilistic bindings as feature units, ChIP-GSM then employs logistic regression to predict active regulatory elements. Validation of ChIP-GSM predicted regulatory regions on multiple independent datasets sharing the same context confirms the advantage of using TF modules for predicting regulatory activity. In a case study of K562 cells, we demonstrate that the ChIP-GSM inferred modules form as groups, activate gene expression at different time points, and mediate diverse functional cellular processes. Hence, ChIP-GSM infers biologically meaningful TF modules and improves the prediction accuracy of regulatory region activities.

## Author summary

Investigating TF binding to different types of regulatory regions can help reveal underlying activation mechanisms. However, accurately inferring modules among a large set of TFs is challenging due to the existence of weak, noisy, and context-sensitive binding signals. To reliably infer TF modules, here we describe ChIP-GSM, a Gibbs sampler built upon a Bayesian framework, that can further predict active regulatory elements. A comparison with other methods demonstrates ChIP-GSM’s improved performance on module identification and active regulatory element prediction. Experimental results demonstrate that TF modules identified by ChIP-GSM are likely mediating distinct cellular functions by activating regulatory regions at different time points.

## Introduction

DNA-binding proteins like transcription factors (TFs) usually function coordinatively as a module, a molecular complex that binds at cis-regulatory regions to modulate the expression of nearby genes. These modules consist of both master factors and mediators [1,2]. Master factors recruit different mediators to activate different types of regulatory regions and their cooperation may change in different contexts [3]. Joint analysis of multiple TFs ChIP-seq profiles have demonstrated the power to recover cell-type-specific regulatory modules [4–9]. ChromHMM [4] inferred chromatin states along the whole genome by modeling the presence or absence of histone marks using a multivariate hidden Markov model. jMOSAiCS [5] inferred combinatorial patterns of TF enrichment at each genomic region by modeling their ChIP-seq read counts using negative binomial mixture distributions. SignalSpider [6] modeled ChIP-seq read coverage using Gaussian mixture distributions and decipher the combinatorial TF binding events in a layered hierarchical probabilistic framework. In these methods, the input ChIP-seq data, which play a key role in modeling background regions and have been demonstrated important for accurate TF binding events identification [10–13], however, were not used, so the amplified background regions that could produce high read counts and confound the identification of binding events, especially weak ones, were not specifically modeled. The false rate on module identification could be high if weak binding events were included in their analysis. Among hundreds of TFs, there are more coactivators than master factors, and little is known about their associations. We need an approach to infer TF modules efficiently and accurately in a wide range of applications.

Here we present ChIP-GSM, a ChIP-seq data-driven Gibbs Sampler to infer Modules (ChIP-GSM) using a Bayesian framework that integrates ChIP-seq profiles of multiple TFs. Using ChIP-seq read counts as input, for each genomic region ChIP-GSM estimates the binding potential of a TF module by jointly modeling ChIP-seq read counts of associated TFs in that module. Specifically, given a TF ChIP-seq profile, ChIP-GSM models its distribution of read counts as a mixture of Power-Law and Gamma distributions [14,15], at TF-bound and background regions, respectively. Using Gibbs sampling, which is designed to sample one parameter at a time to draw samples from a high dimensional probability distribution, ChIP-GSM iteratively evaluates TF-region binding potential, samples ChIP-seq read counts, and probabilistically draws TF module samples for each region. With many rounds of sampling, every candidate module will be sampled and evaluated for the binding occurrence at individual regions. ChIP-GSM will ultimately generate a region-specific sample distribution of all modules. A region can be bound by one or multiple modules as determined by the sample distribution. Across regions in the whole genome, the accumulated samples of each module represent its abundance in the current tissue or cell type.

We compared ChIP-GSM against other approaches [5, 6] that can infer regulatory modules at genome-wide locations using TF ChIP-seq profiles as the input. Using both simulated data and ENCODE ChIP-seq data, we successfully demonstrated that ChIP-GSM predicted a wide variety of modules more accurately, especially those with many weakly bound TFs, than the comparable methods. Further applying ChIP-GSM respectively to enhancers and promoters, we found significantly different associations of the same TFs between these two categories of regions, suggesting that one should infer modules using different models for enhancers and promoters.

Existing data show that transcriptional activities of regulatory regions correlated with binding signals of epigenetic marks [4] and TFs [16]. Here we further demonstrated that ChIP-GSM-inferred TF modules better predict enhancer or promoter transcriptional activities. To achieve this, we trained a logistic regression classifier [17] by combining, in the same context, the learned weighted bindings of TF modules to regulatory regions with the FANTOM5 regulatory region activities (CAGE data) [18]. Using the trained classifier, we predicted systematically the activities of hold-out regions for each of nine select cell types. Results showed that ChIP-GSM (featuring TF modules) performed better than methods that use histone proteins, TFs, or both as feature units [4,16]. Moreover, the top-predicted active regions were significantly more enriched with enhancer or promoter marker signals than the annotated regions in the FANTOM5 database, revealing the refinement on context-specific active regulatory region identification using ChIP-GSM predictions.

Finally, we conducted a case study analyzing the target genes regulated by ChIP-GSM-identified TF modules in K562 cells. We clustered TF modules into groups based on the similarity of TFs between modules and checked the target genes and their expression mediated by each module group. Time-course analysis using a K562 gene expression dataset [19] revealed that genes co-regulated by modules from the same group are significantly co-expressed and involved in similar cellular processes associated with leukemia development. Hence, ChIP-GSM infers biological meaningful TF modules that play important roles in modulating gene expression and mediating functional cellular processes.

## Results

### ChIP-GSM framework

**Fig 1:**
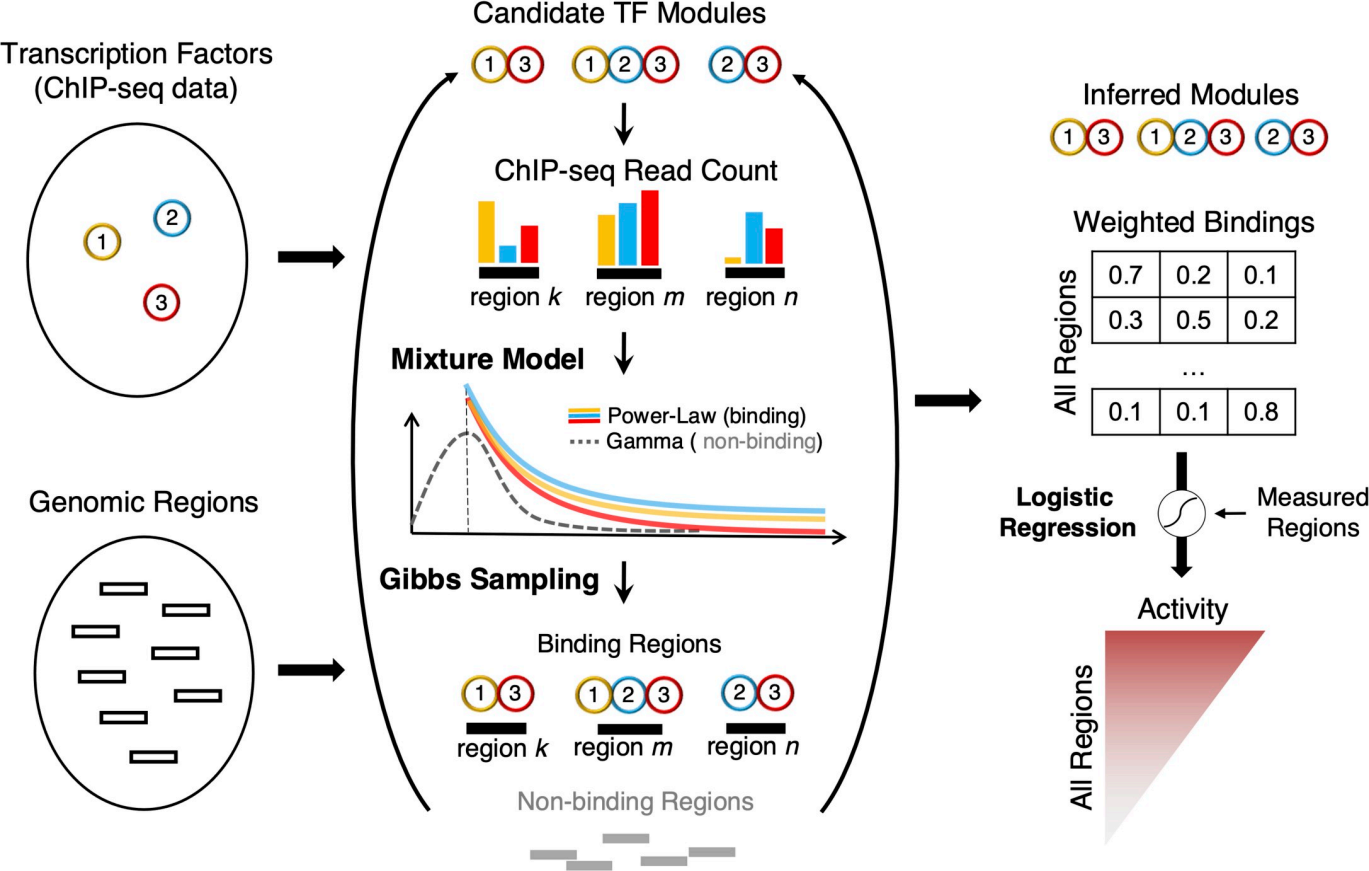
A flowchart of ChIP-GSM for TF module inference. Given ChIP-seq data of multiple TFs and candidate genomic regions, ChIP-GSM learns a mixture model of Power-Law (for binding events) and Gamma (for non-binding events) distributions that best explains the read counts in TF-bound and background regions. ChIP-GSM’s Gibbs sampler iteratively samples TF modules for each region until convergence toward a posterior probability distribution of modules for all regions. Using logistic regression, ChIP-GSM correlates TF module binding likelihoods at individual regions with experimentally measured regulatory activities to systematically predict activities for each region with TF module regulation.

Given ChIP-seq data and candidate genomic regions (regions enriched with ChIP-seq signals and/or with regulation annotations), ChIP-GSM infers TF modules for each region using a Gibbs Sampler and then uses an elastic-net classifier to predict regions transcriptional activities (**Fig 1**). To integrate ChIP-seq data of multiple TFs, we partition the candidate genomic regions into fixed-length bins (i.e., 500 bps long) and calculate the normalized ChIP-seq read count for each TF in each bin using HOMER [20]. ChIP-GSM focuses on regions that are likely to be regulated by a module with more than ten ChIP-seq reads observed from each of at least two TFs.

A probabilistic Power Law-Gamma mixture model is fitted for each pair of TF and control ChIP-seq profiles to respectively model read count distributions of TF-bound and background regions. For each TF, a Power-Law model is fitted to regions with ChIP-seq read counts larger than ten and at least two-fold increase to the read counts in the matched input ChIP-seq profile, as only such regions are likely to be bound by the TF. Background regions that contain amplified open chromatin regions can also have high read counts in a TF ChIP-seq profile [21]. These regions will confound the correct identification of TF-bound regions, especially regions with weak binding events. Therefore, we include a background Gamma model to facilitate the differentiation of TF-bound regions from background regions. This Gamma model is fitted to read counts in the input ChIP-seq profile to learn the distribution features of background regions.

Given multiple TFs ChIP-seq profiles, ChIP-GSM uses a Gibbs sampler to sample TFs iteratively as binding or non-binding according to the Power-Law/Gamma mixture model and estimate the binding potential of each module (a combination of TFs) at individual regions. Specifically, we develop a weight-based read tag tossing approach to identify binding events for multiple TFs simultaneously, where any ‘weak’ binding event with a relatively low read count can be well captured by assigning a weight much higher than that of a background region. These ‘weak’ bindings will help identify the complete association of TFs across regions and further highlight their cooperative action in a tissue or cell type. Once the Gibbs sampler appears to converge on the equilibrium distribution, ChIP-GSM accumulates samples that probabilistically define modules for each region. One region can be bound by one or multiple modules as determined by the modes of the sampling distribution. The accumulated samples of a module across all regions represent its abundance in the current regulatory context. Finally, ChIP-GSM correlates the predictive probabilities of modules with the experimentally measured activity at labeled regions using elastic-net logistic regression and systematically predicts activity of every region. A detailed workflow of ChIP-GSM is provided in **S1 Fig** and **S1 Text**.

### ChIP-GSM accurately identifies TF modules across genome-wide locations

To infer modules among a small number of TFs (e.g., 4), ChIP-GSM performed an exhaustive search of all possible TF combinations. For benchmarking we used ENCODE H1-hESC cell ChIP-seq data for four proteins: EZH2, SUZ12, H3K27me3, and H3K4me3. EZH2 and SUZ12 are Polycomb-group (PcG) proteins [22] and often co-bind to ‘bivalent’ domains marked by H3K27me3 and H3K4me3 [23,24]. Given strong associations between the four proteins, a high abundance (# of regions with a TF module / # of all regions) for a full module of all four factors was expected. We compared ChIP-GSM to two approaches: jMOSAiCs featuring a negative binomial model and SignalSpider featuring a Gaussian mixture model. Totally, ChIP-GSM identified 6,564 regions regulated by a module of all four proteins, with an abundance of 29%, while the other comparable methods like SignalSpider [6] and jMOSAiCs [5] identified fewer such regions, with the module abundance of 21.6% and 9.65%, respectively.

**Fig 2:**
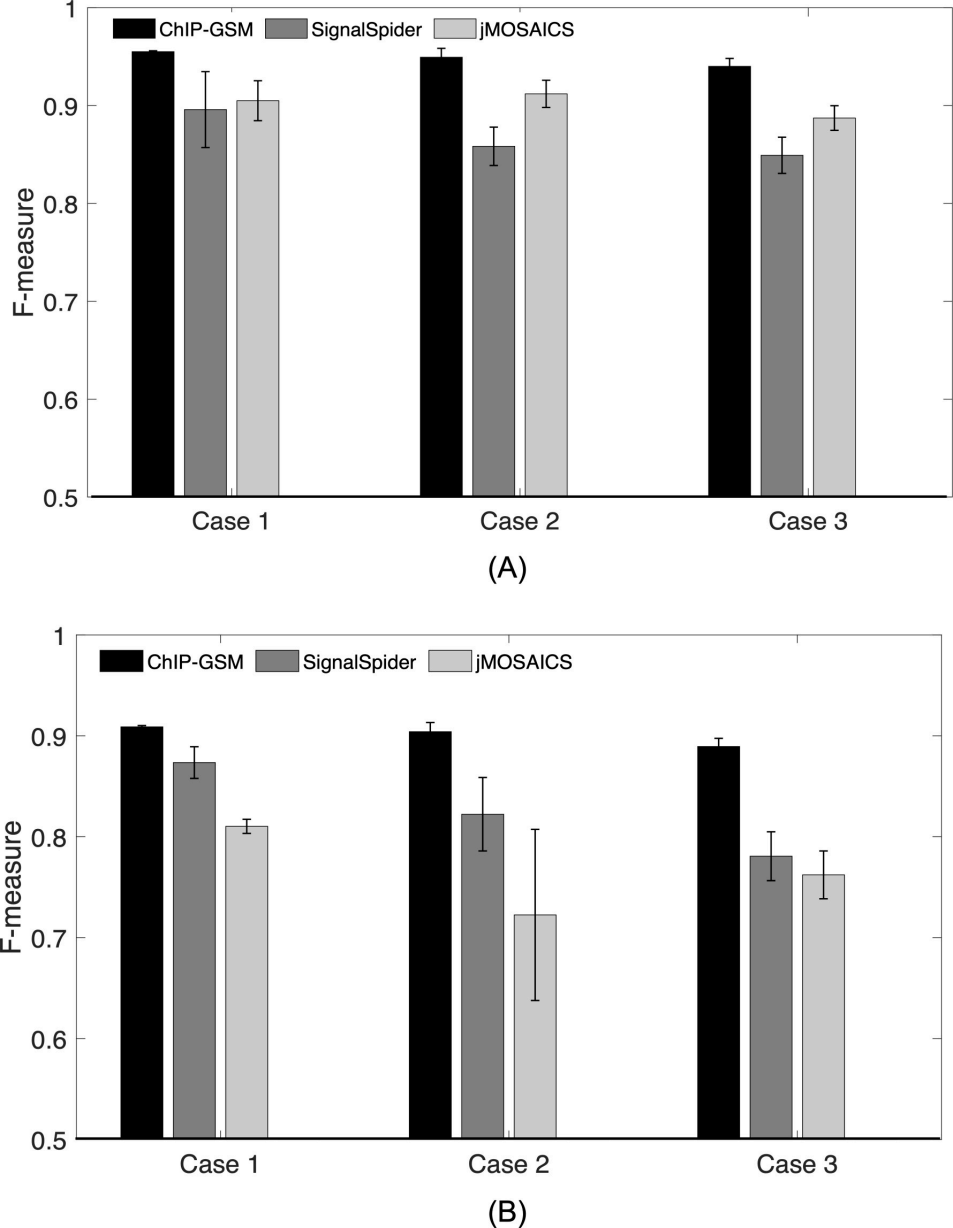
ChIP-GSM and competing methods abilities to infer TF modules using realistically simulated ChIP-seq data. We simulate ChIP-seq read counts for 100,000 regions and examine the accuracy of module inference by applying each competing method to a low challenging case (Case 1, four TFs), a middle challenging case (Case 2, seven TFs) and a high challenging case (Case 3, eighteen TFs). (A) F-measure of each method on module inference across all regions; (B) F-measure of each method on regions with at least one weak binding event. ChIP-GSM performs better than the comparable methods, especially when there are many TFs with weak binding events.

To quantitively evaluate ChIP-GSM’s accuracy, we simulated genome-wide read counts for four proteins based on the real ChIP-seq data (with real TF combinations retained to individual regions) [14] and generated 10 replicates with random noise read counts added to individual regions. ChIP-GSM and comparable methods were applied to the simulated data under the default settings for each method. To fairly compare performances of competing methods, we evaluated for each model the accuracy on TF-region binding events identification. Then, binding events in incompletely identified modules were still counted as the competing methods might miss weak binding events. To account for both false positive and false negative rates, we calculated the F-measure (= 2/(1/precision+1/recall)), for binding events at all regions (**Fig 2A,** Case 1) or at regions containing at least one ‘weak’ binding factor (**Fig 2B,** Case 1). The F-measure of ChIP-GSM was 0.96 for all regions, higher than the best performance of the competing methods. Although ChIP-GSM performed an exhaustive search of all possible TF combinations for each region, due to the ChIP-seq signal noise and possibly imperfect separation of some weak bindings from background regions, the F-measure was not 1. For regions with weak binding events, ChIP-GSM maintained its accuracy around 0.9 while the competing methods dropped their performance to 0.8.

When the number of investigated TFs grows, the TF-TF cooperation becomes complex and computationally intractable for an exhaustive search over all possible TF combinations. Therefore, In large-scale applications, for example, inferring modules from more than 10 TFs, ChIP-GSM first performs a primary search of candidate modules based on the frequency of TF co-colocalizations across the whole genome and then efficiently identifies regions bound by each candidate module. To evaluate the performance of ChIP-GSM in such cases, we designed realistic simulations based on real ChIP-seq profiles. Our design had three advantages as: (1) each ChIP-seq profile was unique in binding/non-binding locations and read count distributions; (2) it had both strong and weak binding events (to simulate TFs with a range of binding strengths); (3) real associations among TFs were largely retained at the original locations (not using artificial combinatorial patterns and assignments to random locations). We simulated two scenarios: Case 2 with a medium number of seven TFs and Case 3 with a high number of eighteen TFs. As can be seen from **Fig 2** that in both cases, ChIP-GSM performed the best among all competing methods, especially for module inference at regions with weak bindings. The primary search of candidate modules would miss some rare TF combinations and their associated regions; thus, the performance of ChIP-GSM indeed degraded, but overall, it was comparable to the performance of exhaustive search. These results demonstrated that the Power-Law/Gamma mixture model proposed in ChIP-GSM worked better on identifying module-bound regions than the negative binomial model used in jMOSAiCs or the Gaussian mixture model used in SignalSpider.

### ChIP-GSM infers TF modules specific to enhancers or promoters

**Fig 3:**
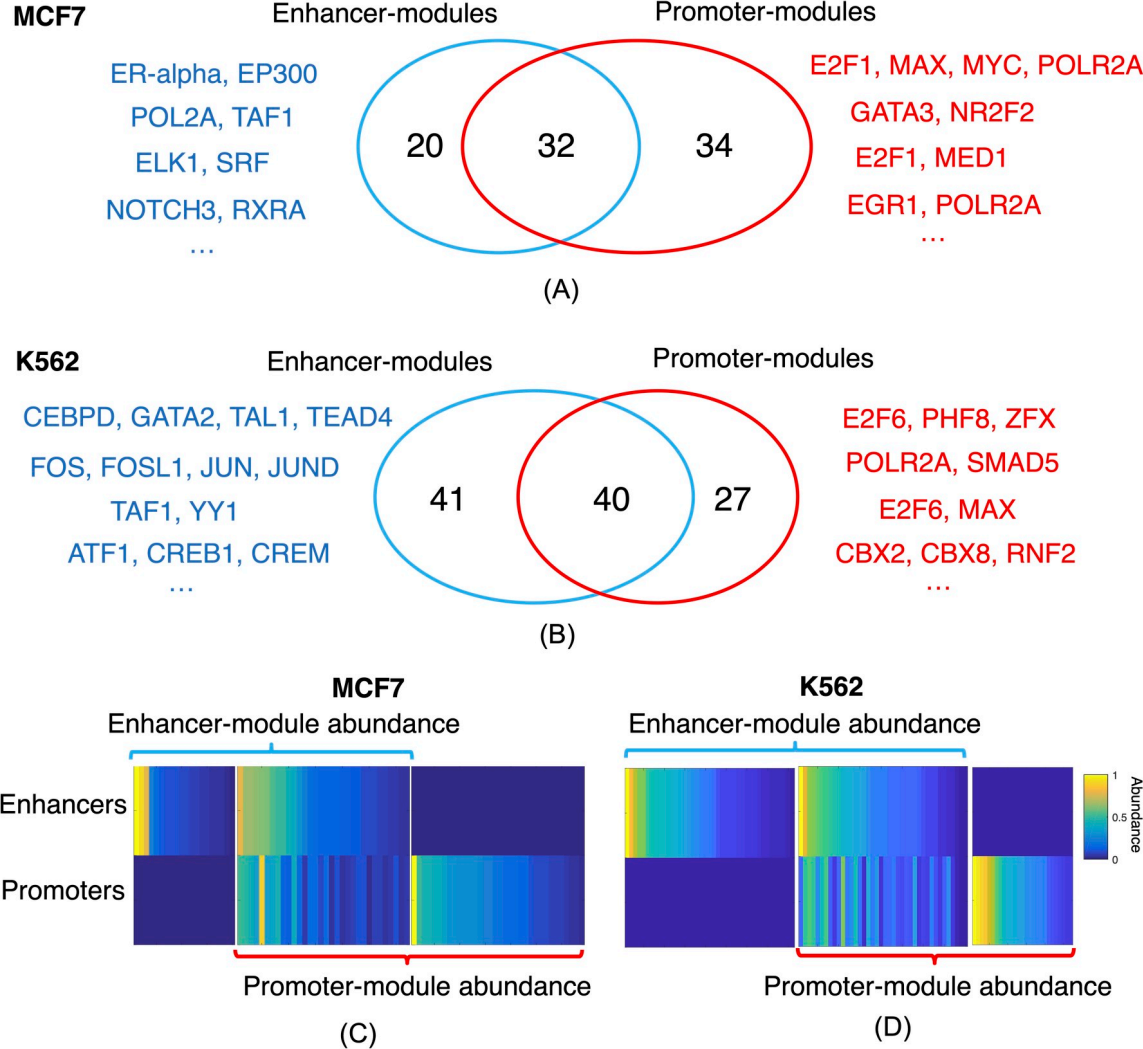
ChIP-GSM-inferred TF modules for enhancer and promoter regions respectively. The number of modules functioning at enhancer or promoter regions in (A) MCF-7 cells or (B) K562 cells. Module abundance reveals that region-specific modules can be as strong as common modules functioning in both enhancer and promoter regions, in (C) MCF-7 cells or in (D) K562 cells.

It has been known that epigenetic marks at enhancers differ from those at promoters [25,26], e.g., H3K27ac for enhancers and H3K4me3 for promoters. For these well-annotated regulatory regions, we further studied the difference of TF-associations at these two types of regions using ChIP-seq data from nine different cell types including breast cancer MCF-7 cells, leukemia K562 cells, and other major cell types that have a sufficient number of TF ChIP-seq profiles in the ENCODE database (**S1 Table**). For each cell type, ChIP-GSM integrated cell type-specific TF ChIP-seq data from the ENCODE data portal and inferred TF modules at enhancers and promoters, respectively. We found that the mean percentage of TF modules shared between enhancers and promoters across nine cell types was only 47% (**S2 Table**), revealing the big difference in the modulization of TFs at these two types of regions. Further analysis of module abundance showed that enhancer- or promoter-specific modules can be as strong as common modules (**Fig 3B** and **3D**). Identified modules for each cell type are provided in **S3 Table.**

For breast cancer MCF-7 cells, ChIP-GSM identified 52 enhancer-modules and 66 promoter-modules, with a similarity of 54% (**Figs 3A** and **S2** and **S3 Table**). The top two enhancer-modules were ERalpha-EP300 and POL2A-TAF1 (listed in **Fig 3A**). Both associations were well supported by their known functions as: EP300 was a signature protein of enhancers; ER-alpha was a major enhancer activator in MCF-7 cells [27]; TAF1- POLR2A was also an enhancer signature [28]. The top promoter-module was E2F1-MAX-MYC-POLR2A. The association of MYC, MAX, and POLR2A was frequently found at both enhancers and promoters in MCF-7 cells [29], but E2F1 bound more often to promoters [30]. Consistent with this finding, ChIP-GSM identified a promoter-module containing the promoter-specific factor E2F1 and a common submodule MAX-MYC-POLR2A between enhancers and promoters.

For K562 cells, ChIP-GSM identified 81 enhancer-modules and 67 promoter-modules, with a similarity of 55% (**Figs 3C** and **S2** and **S3 Table**). A strong association was highlighted among CEBPD, GATA2, TAL1, and TEAD4 at enhancers. TAL1, TEAD4, and CEBPD were all master transcription factors with binding signals enriched at super-enhancers [2], GATA2 was prevalent at dynamic enhancers [31], and a similar module was previously found at K562 enhancer regions in an independent study [32]. At promoter regions, the top-predicted module was E2F6-PHF8-ZFX. E2F6 binding sites were demonstrated to be proximal to TSSs [33] and PHF8 usually bound to H3K4me3-enriched regions, also close to TSSs [34]. ZFX was reported to be binding at CpG island promoters in many tumor cell types [35].

### ChIP-GSM improves the prediction of regulatory region activity

Regulatory regions usually harbor histone mark enrichments and are primarily activated by a molecular complex of TFs. ChIP-GSM, which models TF modulization at regulatory regions, could further improve the prediction performance on active regulatory regions than methods using individual histone marks or TFs as feature units. To explore these, we used FANTOM5 CAGE data [18] as experimental measures of the annotated enhancers activities and studied the prediction performance of ChIP-GSM results on these regions in a supervised framework. Specifically, for each cell type, an enhancer region was labeled as ‘positive’ if its eRNA TPM value was larger than 1 in at least two FANTOM5 CAGE samples of the current cell type; labeled as ‘negative’ if it was inactive in the current cell type but active in others. Similar labeling was done for promoter regions based on the genes mRNA expression.

For each cell type, we trained an elastic net logistic regression classifier by combining ChIP-GSM TF module probabilistic estimations with 80% labeled active/inactive regulatory regions. Features were ChIP-GSM inferred probabilities of all TF modules binding to individual regions. Here, ChIP-GSM parameters like PowerLaw-Gamma distribution parameters and thye candidate TF combinations were fitted only using data from training regions (**S4 Table**). Feature values (module probabilities) between training and testing regions were independent as the modules were sampled and evaluated individually and independently at different regions (**Methods**). For performance comparison, we included three supervised approaches: EMERGE [16], an elastic net model using as input all cell type relevant histone marks and TF ChIP-seq profiles; MatchedFilter [25], a linear SVM model with matched-filter scores of discriminative epigenetic marks H3K27ac, H3K4me1, H3K4me2, H3K4me3, H3K9ac and DHS; and a Baseline model using as input features of TF binding profiles (i.e., peaks from ENCODE).

**Fig 4:**
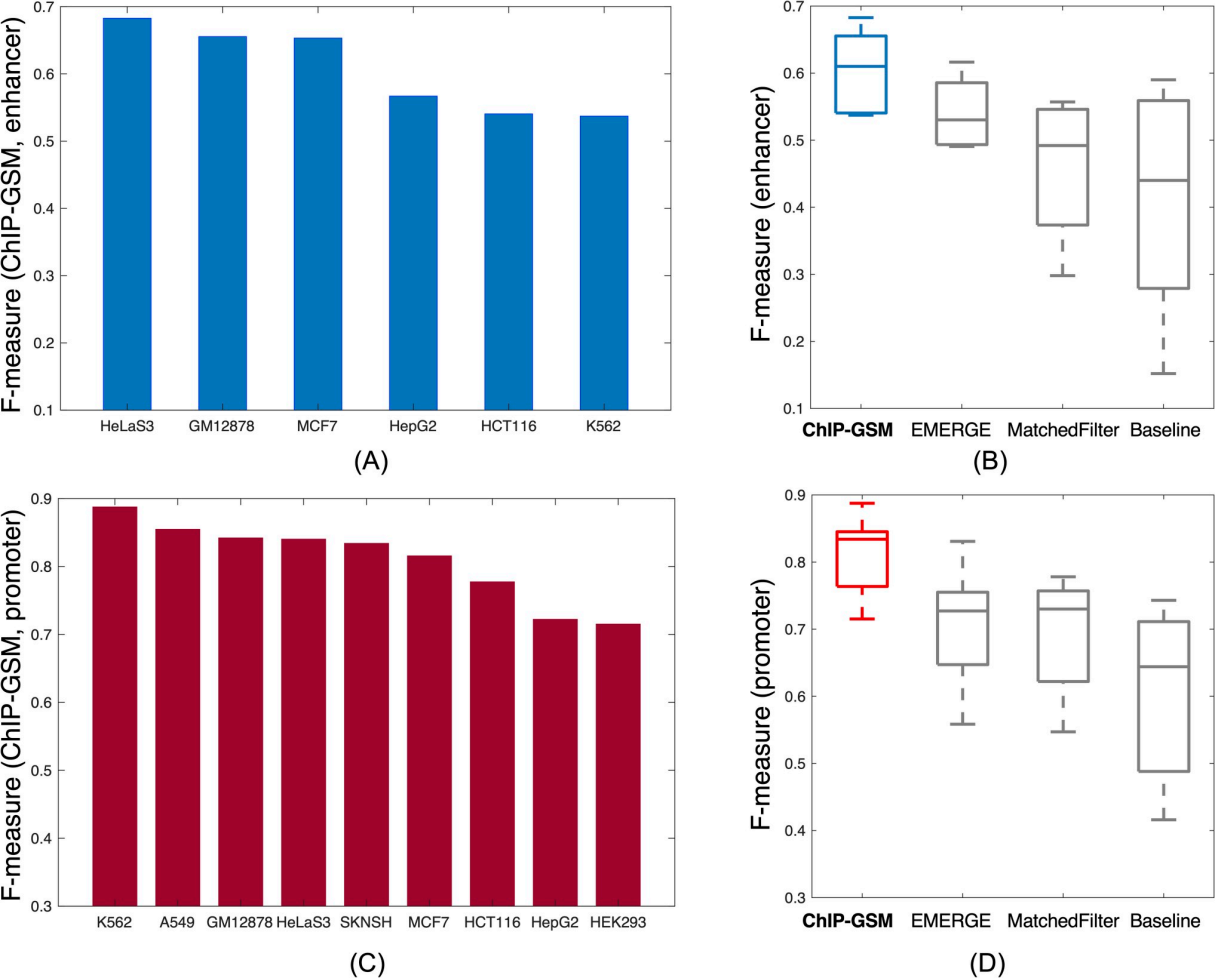
Improved ChIP-GSM prediction of cell type-specific active enhancers and promoters. (A) and (C) show the F-measure of ChIP-GSM on the 20% hold-out labelled enhancers or promoters. (B) and (D) show boxplots of F-measures of ChIP-GSM and three comparable methods across all cell types.

We applied the above methods to nine cell types and for each cell type compared their prediction accuracy (F-measure) on the 20% hold-out regions. ChIP-GSM indeed performed better at predicting active regulatory elements than did methods that used histone marks, transcription factors, or both as feature units (**Fig 4** and **S5 Table**). For each cell type, the baseline model using TF binding signals performed similarly to MatchedFilter, a method featuring epigenetic marks, though requiring considerably fewer input files. EMERGE combined TF and HM binding signals and got a higher prediction accuracy but it still performed worse than ChIP-GSM. In the more challenging test where only 50% of regions were used during the module learning and classifier training process, ChIP-GSM still had a higher prediction accuracy than EMERGE (**S2 Fig**).

**Fig 5:**
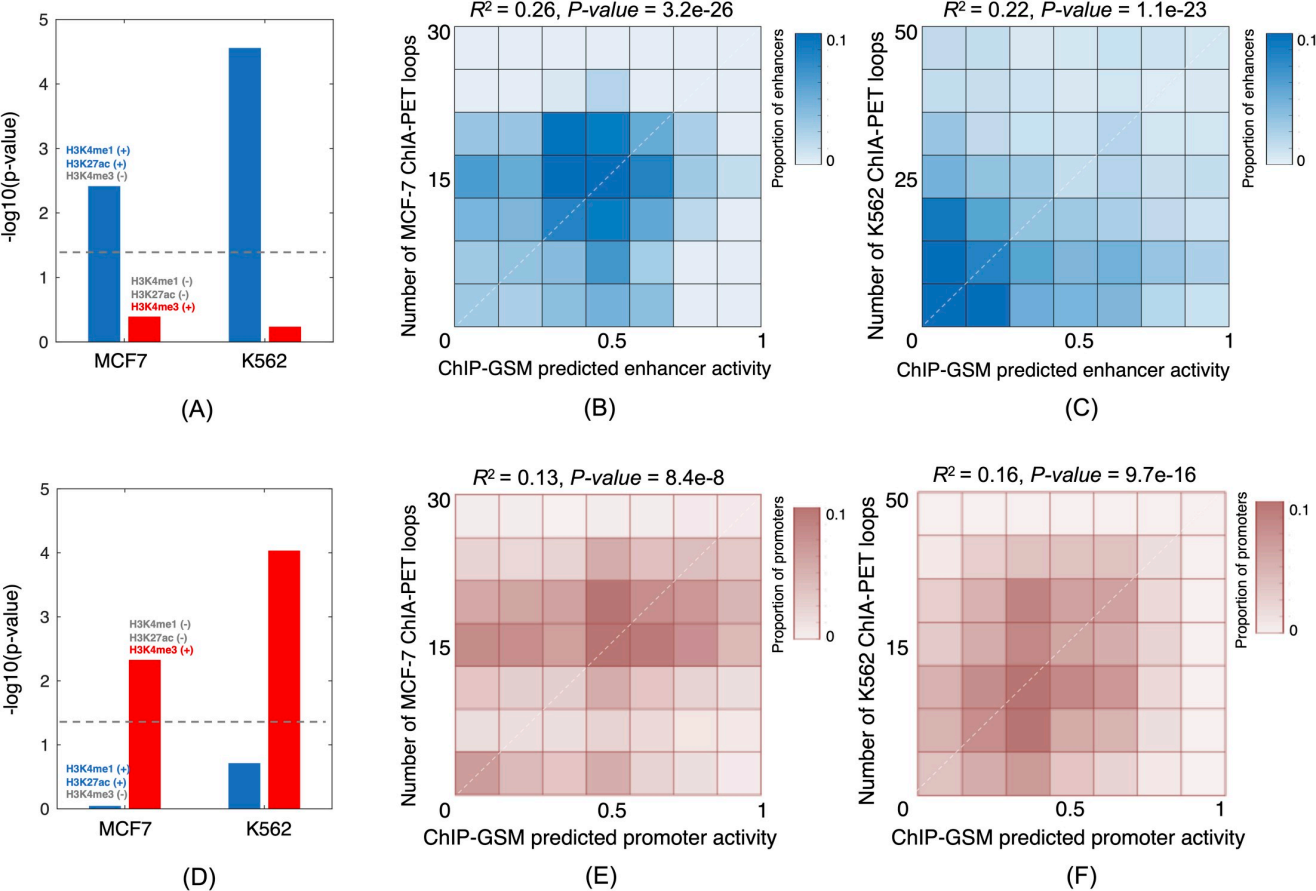
ChIP-GSM-predicted active regions are significantly enriched with epigenetic markers and significantly correlated with 3D chromatin interactions. (A) The top 10% predicted enhancers are significantly enriched with marker ChIP-seq peaks of H3K4me1 and H3K27ac but not H3K4me3. (B) and (C) The ChIP-GSM-predicted enhancer activities are significantly correlated with ChIA-PET 3D chromatin interactions in MCF7 and K562 cells, respectively. (D) The top 10% of predicted enhancers are significantly enriched with marker peaks of H3K4me3 but not H3K4me1 or H3K27ac. (E) and (F) The ChIP-GSM-predicted promoter activities are significantly correlated with ChIA-PET 3D chromatin interactions in MCF7 and K562 cells, respectively.

We trained a classifier using all labeled regions and reprioritized all regions based on the ChIP-GSM predicted activities. We validated the top predictions by assessing the enrichment of enhancer markers H3K4me1 and H3K27ac or promoter marker H3K4me3 [26]. ChIP-seq peaks for each marker were downloaded from the ENCODE database. Taking MCF-7 and K562 cell types for examples, compared to the active enhancers reported in the FANTOM5 database, the top 10% of the ChIP-GSM-predicted enhancers were significantly more enriched with ChIP-seq peaks of H3K4me1 (+), H3K27ac (+) and H3K4me3 (-) (**Fig 5A**, *p*-values = 2.76e-5 for K562 and 3.83e-3 for MCF-7; **Methods**). The top 10% ChIP-GSM-predicted promoters were also significantly more enriched in peaks with H3K4me1 (-), H3K27ac (-) and H3K4me3 (+) than did FANTOM5 active promoter regions (**Fig 5D**, *p*-values = 9.23e-5 for K562 and 4.7e-3 for MCF-7). These results suggested that active regulatory regions can be further refined by combining their activity measurements with TF modules binding there.

Furthermore, it has been reported that the activities of enhancers or promoters are highly associated with nearby 3D chromatin interactions [36,37]. Consistent with this notion, for cell types MCF7 and K562 we examined the correlation of ChIP-GSM-predicted activities and the number of nearby ChIA-PET interactions. Shown in **Fig 5** are smoothed scatter plots represented as matrices, where each cell is colored proportional to the number of data points where the x- and y-axes correspond to the ChIP-GSM predicted activity and the number of ChIA-PET loops, respectively. For the MCF-7 cell type, the ChIP-GSM-predicted activity was significantly correlated with the number of ChIA-PET interactions (**Fig 5B** and **5E**, *p*-values = 3.2e-26 for enhancers and 8.4e-8 for promoters). Similarly, significant results were found for the K562 cell type, too (**Fig 5C** and **5F**, *p*-values = 1.1e-23 for enhancers and 9.7e-16 for promoters). Raw scatter plots were provided in **S3 Fig**, from which we can also see that regions with higher scores predicated by ChIP-GSM were actively interacting with more nearby regions through the 3D genome folding.

### ChIP-GSM inferred modules mediate diverse cellular functions in K562 cells

**Fig 6:**
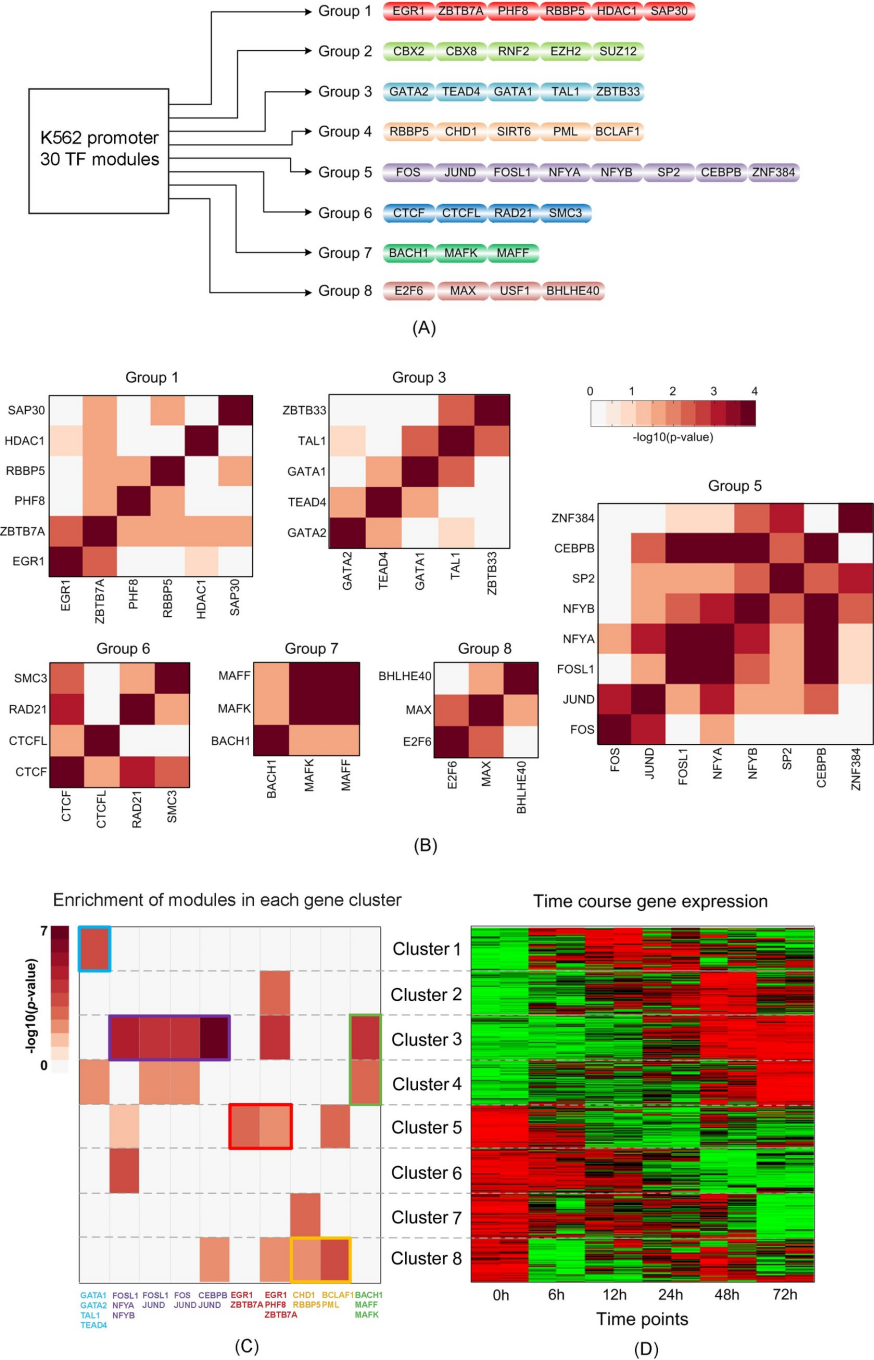
ChIP-GSM-identified TF modules at the gene promoter regions of K562 cells. (A) Eight groups of modules identified by ChIP-GSM functioning at gene promoter regions in leukemia K562 cells (TF modules are defined in **S4 Fig**); (B) mRNA co-expression of pairwise TFs in each group; (C) selected modules whose target genes are significantly enriched (hypergeometric p-value < 0.001) in activated genes as shown in (D). Each color label or box represents a unique module group.

Finally, we studied target genes of ChIP-GSM-inferred modules and further, cellular functions that they regulated. Here, we selected K562 promoter-modules (as listed in **S4 Fig**) for further analysis because: (1) these modules were most highly predicted to be activating promoters (**Fig 4C**; F-measure = 0.89); and (2) ChIP-GSM identified more high-resolution, biologically important modules (**S4 Fig**) than other methods for inferring modules from a large number of TFs (**S5–S7 Figs**). In total, these modules were clustered into eight non-overlapping groups based on the TFs they shared in common (**Fig 6A**). Gene expression and functional analysis of these TFs showed that TFs in each module group are highly co-expressed [19] (GEO access number: GSE1036) and shared coherent functions (**Figs 6B** and **S8** and **S2 Text**), thereby supporting the notion that cooperative TFs were typically activated simultaneously [38, 39].

Gene set functional enrichment analysis (using DAVID [40]) showed that these module groups regulate diverse cellular functions associated with leukemia development. And each group tend to mediate cellular functions different from those of other groups (**S9 Fig** and **S6 Table**). For example, Group 1 TFs regulated genes significantly associated with acute myeloid leukemia; Group 4 TFs regulated genes that were involved in cancer development, including chronic myeloid leukemia; Group 5 TFs regulated genes significantly associated with cell survival-related functions including cell death, apoptosis, and p53 signaling. These results suggested that TFs in one group likely regulated specific cellular processes than did TFs in the other groups.

To examine if the identified TF module groups were active at different cell stages, we checked the target gene expression (**Fig 6D**). Totally we collected 1,569 genes that are actively expressed in K562 cells [19]. Based on their time course expression pattern, we clustered genes into eight clusters (Clusters 1–8) and for each cluster, we assessed its enrichment of target genes regulated by each module. Significant gene cluster-TF module pairs (adjusted hypergeometric *p*-value < 0.001) are shown in **Fig 6C** (boxed in colors that match the colors of the module groups in **Fig 6A**; gene symbols are listed in **S7 Table**). We found that modules from different groups rarely functioned at the same time. For instance, one module from Group 3 (labeled by the ‘cyan’ box) was highly enriched in Cluster 1. Modules (labeled by the ‘purple’ box) from Group 5 and their target genes were significantly enriched in Cluster 3. Modules from Group 4 (labeled by the ‘yellow’ box) did not share many genes, but both were enriched in Cluster 8. These observations suggested that our identified module groups regulated genes at different cell stages of K562 cells and mediated specific cellular functions associated with leukemia development.

## Discussion

Inferring modules among many TFs is challenging because TF binding signals are diverse, noisy, and sensitive to the cellular environment. A benefit of this analysis, however, is that it helps explain regulatory region activation and target gene expression. ChIP-GSM probabilistically predicts TF modules and active regulatory elements based on ChIP-seq read counts; consequently, it can help characterize modules that are associated with regions containing weak, potentially cell-type-specific binding signals (which can be easily missed by peak callers). Moreover, we demonstrate that TF modules are better than TF peaks or histone modifications at predicting active regulatory elements. As a general computational framework, ChIP-GSM can infer modules from genome-wide locations with ChIP-seq read coverage of at least two TFs, not limited to annotated regulatory regions, although applications to known regions would allow better results interpretation and validation.

Using the cell type K562 as a case study, we demonstrate the functional diversity of ChIP-GSM inferred modules: target genes of different modules are actively expressing at different time points and regulating distinct cellular processes. We anticipate that TF modules at enhancer regions have similar functional diversity. Currently, however, the sparsity of high-resolution chromosome interaction data, such as ChIA-PET, obscures the association between enhancers and genes, especially for enhancers that are distantly located from the genes they regulate.

Because most ChIP-seq profiles are generated using cell line models, we mainly applied ChIP-GSM to cell line ChIP-seq data. ChIP-GSM can also be applied to ChIP-seq data from human tissues, for which the binding signals are much noisier and exhibit more variable binding strengths than *in vitro* cell lines, which may limit ChIP-GSM’s accuracy. Major host databases, like ENCODE, are periodically updated by replacing low-quality samples with new, high-quality replicates. The ChIP-GSM code is publicly accessible (https://sourceforge.net/projects/chipgsm/) so that users can apply it to the newest release of ENCODE or newly published high-quality ChIP-seq data. Hence, ChIP-GSM has the potential to uncover more detailed TF associations overlooked by conventional methods, thereby leading to new biological insights relevant to human disease.

## Methods

### ChIP-GSM: TF module inference

#### Candidate TF module searching

The computational complexity of TF module inference (studying associations between TFs) increases exponentially with the number of TFs (*T*): for a large set of TFs, exploring exhaustively all possible combinations is intractable (e.g., for *T* = 50 there will be 2^50^ combinations). Consequently, ChIP-GSM first identifies a list of candidate modules based on the number of TFs and their ChIP-seq read counts across all regions. In detail, if the number of TFs is no larger than 6, ChIP-GSM will perform an exhaustive search using all TF combinations as candidate modules. This is suitable in small-scale studies with selected ChIP-seq data of closely related TFs (see the study of Polycomb-group proteins in H1-hESC cells and simulation). If the number of TF is larger than 7, ChIP-GSM will primarily search for a candidate list of TF modules. Specifically, for each TF ChIP-GSM roughly identifies its binding sites as regions with a read count larger than 10 and a fold change to the input read count larger than 2. Then it selects the top 100 TF combinations that regulate the most regions as candidate modules. This is suitable in large-scale studies (see the study of MCF-7 and K562 cell types). The candidate modules are stored in matrix **B,** with *M* rows (the total number of candidate modules) and *T* columns (the total number of TFs), where each row is a binary vector [*b*(*m*, 1), *b*(*m*, 2),…,*b*(*m*, *t*),…,*b*(*m*, *T*)] representing a candidate TF combination. To model background regions that are not regulated by any module, we add an all-zero row (*m* = 0) to **B**.

#### Read count modeling

Given a total of *K* regions, for each region *k* we define a module index variable *c*_*k*_, with *c*_*k*_∈[1…*m*…*M*] if this region is regulated by a candidate module or *c*_*k*_ = 0 otherwise. Further, if *b*(*c*_*k*_,*t*) = 1, the region *k* is a binding region of TF *t*; if *b*(*c*_*k*_,*t*) = 0, the region *k* is a background region. The observed read count *Y*_*k*,*t*_ is fitted to a mixture model as follows:

$$Y_{k,t}=b(c_{k},t)X_{k,t}+(1-b(c_{k},t))I_{k,t}+N_{k,t},$$ (1)

$$X_{k,t}∼\mathrm{PowerLaw}(X_{min},\gamma_{t}),$$ (2)

$$I_{k,t}∼\mathrm{Gamma}(\alpha_{t},\beta_{t}),$$ (3)

$$N_{k,t}∼\mathrm{Gaussian}(0,\sigma_{N}^{2}),$$ (4)

$$c_{k}∼\mathrm{Uniform}[0,M],$$ (5)

where *X*_*k*,*t*_ represents the read count of a TF-bound region and follows a Power-Law distribution with hyper-parameters *X*_*min*_ and *γ*_*t*_; *I*_*k*,*t*_ represents the read count for a background region and follows a Gamma distribution with mean and shape parameters *α*_*t*_ and *β*_*t*_; *N*_*k*,*t*_, represents the residual between the observed read count and the distribution-fitted read count. A residual at each individual region can be either positive or negative. We assume a zero-mean Gaussian distribution on it to ensure that the mean of probabilistically sampled read counts (*X*_*k*,*t*_ or *I*_*k*,*t*_) across all regions is the same as the mean of observed ChIP-seq read counts (*Y*_*k*,*t*_). To control the scale of residuals of all regions, we use a prior Inverse-Gamma distribution on the variance variable $\sigma_{N}^{2}$. Inverse-Gamma distribution has been widely used to model the posterior distribution for the unknown variance of a normal distribution. Its probability density distribution has a thin tail so that the possibility for $\sigma_{N}^{2}$ to be large is very low. That will ensure that in most cases, the fitted model aligns tightly to the input data.

The mixture model is then applied to each TF ChIP-seq profile for TF-bound/background determination at each genomic location. With *X*_*min*_ = 10 as a hard cut-off, any regions with read counts less than 10 will not be evaluated for binding occurrence. Under this setting, as shown in **S10 Fig**, the Power-Law distribution fits well the real ChIP-seq count data at TF-bound regions. Background regions with high read counts will likely confound the identification of true but weak binding events. Therefore, for background regions, we turn the Gamma distribution parameters to better fit the high read count regions from the input ChIP-seq profile, with the underestimation mostly on regions with read counts less than 10 (**S10 Fig**), because these regions are always treated as background regions (with probability = 1), independent from their fitted probabilities. Learned ChIP-GSM model parameters for cell-type-specific ChIP-seq signals at promoters or enhancers are provided in **S4 Table**. Variable *N* is used to control the residue between the fitted model and the observed data. We control its variance using an inverse Gamma distribution during the sampling process to ensure that the estimated read counts by our model overall fit the observed raw values tightly. Using simulation studies we demonstrate that this Power-Law/Gamma mixture model works better on identifying TF-bound regions than the negative binomial mixture model [5] or the Gaussian mixture model [6] (**Fig 2**).

For TF-bound regions at gene promoters, previous studies reveal that there is an exponential decay effect on read enrichment along with the increase of relative distance to the nearest transcription starting site (TSS) [10,41]. For background regions, the distribution of read enrichment is usually uniform. At enhancers, because both TF-bound and background regions are distal to TSSs, the regulatory effects are independent of their binding locations (due to the loop structure between enhancers and target genes). To better identify TF-bound regions near the TSS, we model the effects of a regulatory region on target gene using the distance-based mixture model:

$$\left\{ \begin{array}{l} P(d_{k}|b(c_{k},t)=1)∼\lambda_{t}exp(-\lambda_{t}|d_{k}|)\\ P(d_{k,t}|b(c_{k},t)=0)∼{\Delta d/d}_{p} \end{array},\right.$$ (6)

where *d*_*k*_ represents the relative distance of region *k* to the nearest TSS; *λ*_*t*_ is the exponential decaying parameter; Δ*d* represents the region length (500 bps); *d*_*p*_ represents the length of the promoter around the TSS (20k bps: +/- 10k bps around TSS). Here, parameter *λ*_*t*_ is TF-specific and estimated from the relative distance distribution of TF *t* binding regions.

For all *K* regions, given the observed ChIP-seq read counts (**Y**), candidate modules (**B**), and the relative binding locations to TSS (**D**), we jointly and iteratively estimate module indexes **C** for all regions based on the conditional probability:

P(C,X,I|Y,B,D)∝P(Y|C,X,I,B,D) (7)

Variables of **X** (read counts of TF-bound regions) and **I** (read counts of background regions) are both dependent on **C**, because the binding status of each TF at each region is determined by the binary binding variable in **B** indexed by **C**. Iterative Gibbs sampling of module variables after reaching equilibrium approximates the following posterior probability distributions:

P(X|Y,C,B,D), (8)

P(I|Y,C,B,D), (9)

P(C|Y,X,I,B,D). (10)

#### Gibbs sampler initialization

To initialize the sampler, ChIP-GSM first calculates *T* weights for each region, corresponding to the binding likelihoods of *T* TFs, where each weight is estimated based on the TF read count *X*_*k*,*t*_ (or *I*_*k*,*t*_) given the binding or non-binding status of the region *k*. *R*_*t*_ denotes the total number of reads for the TF *t* across all *K* regions from its ChIP-seq profile. To calculate the initial weight of each region, ChIP-GSM first roughly estimates the number of reads to be respectively assigned to TF-bound and background regions by simulating the ChIP-seq sequencing process [14]. In detail, we assume that all regions are background and calculate an initial weight *p*_*k*,*t*_ according to the observed read count of the TF *t* at the region *k*. We then select regions with a read count (*Y*_*k*,*t*_) larger than 50 as TF-bound regions and amplify their weight by *F* times (denoting the fold change of a TF-bound region to a background region). The total number of reads aligned to all TF-bound regions is estimated as:

$$R_{X,t}=\frac{R_{t}F\sum_{k}b(c_{k},t)p_{k,t}}{F\sum_{k}b(c_{k},t)p_{k,t}+\sum_{k}(1-b(c_{k},t))p_{k,t}}.$$ (11)

We assign *R*_*X*,*t*_ reads one by one to each of its target regions according to their amplified weights and get an initial read count *X*_*k*,*t*_ for each. Similarly, we assign the remaining reads (*R*_*t*_−*R*_*X*,*t*_) to each of the other background regions according to the initial weights and get a read count *I*_*k*,*t*_ for the region *k*. In general, *X*_*k*,*t*_ or *I*_*k*,*t*_ may differ from the observed ChIP-seq read count *Y*_*k*,*t*_. We aim to minimize this difference across all regions and all TFs by iteratively estimating the TF module variables.

#### Sampling ChIP-seq read counts

To sample the read count for each region, ChIP-GSM updates the weight for each region, assigns reads to them probabilistically and estimates a new read count for each TF. Specifically, for the region *k*, given the observed read count *Y*_*k*,*t*_, the binding state *b*(*c*_*k*_, *t*), and the estimated read count *X*_*k*,*t*_ or *I*_*k*,*t*_, ChIP-GSM calculates a conditional probability as that region’s updated weight:

$$\left\{ \begin{array}{l} {P(X_{k,t}|Y}_{k,t},d_{k},c_{k},B)\propto {P(Y_{k,t}|X}_{k,t},b(c_{k},t)=1)P(X_{k,t})P(d_{k}|b(c_{k},t)=1),\\ {P(I_{k,t}|Y}_{k,t},{d_{k},c}_{k},B)\propto {P(Y_{k,t}|I}_{k,t},b(c_{k},t)=0)P(I_{k,t})P(d_{k}|b(c_{k},t)=0). \end{array}\right.$$ (12)

Each TF-bound region, according to the definition of Power-Law distribution, must contain at least *X*_*min*_ reads. Thus, ChIP-GSM assigns *X*_*min*_ reads evenly to all assumed TF-bound regions and then assigns the remaining reads (*R*_*X*,*t*_−∑_*k*_*b*(*c*_*k*_, *t*)*X*_*min*_) one by one to each of them according to the distribution of their updated weights—leading to each region having a new read count *X*′_*k*,*t*_. Similarly, for background regions, ChIP-GSM assigns *R*_*t*_−*R*_*X*,*t*_ reads one by one to them according to the distribution of updated weights—leading to each background region having new a read count *I*′_*k*,*t*_. Finally, for every region, a probabilistically sampled read count *Y*′_*k*,*t*_ (*b*(*c*_*k*_, *t*)*X*′_*k*,*t*_+(1−*b*(*c*_*k*_, *t*))*I*′_*k*,*t*_) is generated, with a residual *N*_*k*,*t*_ from the observed read count *Y*′_*k*,*t*_. We control the residual variance $\sigma_{N}^{2}$ using an inverse Gamma distribution. The conditional probability of the variable $\sigma_{N}^{2}$ is calculated as follows:

$$P(\sigma_{N}^{2}|Y,X,I,C)\propto \prod_{k,t}{P(Y_{k,t}-Y\prime }_{k,t}|\sigma_{N}^{2})P(\sigma_{N}^{2}).$$ (13)

As detailed in **S3 Text**, Eq (13) is an inverse-Gamma distribution. We directly sample $\sigma_{N}^{2}$ with the updated mean and shape parameters as $\alpha_{N}+\frac{KT}{2}$ and *β*_*N*_+∑_*k*,*t*_(*Y*_*k*,*t*_−*Y*′_*k*,*t*_)^2^/2.

#### Sampling TF modules

For the region *k*, to sample a module from all candidates, ChIP-GSM estimates a discrete probability distribution for all modules by calculating a conditional probability for every candidate module *c*_*k*_ = *m*, and then probabilistically samples a module for the current region:

$$P(c_{k}=m|Y,X,I,D,B)=\frac{\prod_{t}P(Y_{k,t}|{Y^{\prime }}_{k,t})P({Y^{\prime }}_{k,t})P(d_{k}|b(m,t))}{\sum_{j}\prod_{t}P(Y_{k,t}|{Y^{\prime }}_{k,t})P({Y^{\prime }}_{k,t})P(d_{k}|b(j,t))}.$$ (14)

After repeating module sampling at all regions, an updated matrix **C** is obtained and brought back to Eq (11) to initiate a new round of sampling. We run the sampling process until the sampler appears to converge on the equilibrium distribution and then start accumulating samples on TF modules per region. After drawing enough samples, we obtain a weighted matrix $\hat{C}$ with each element ${0\leq \hat{c}}_{k,m}\leq 1$ (sampling frequency) denoting the posterior probability for the module *m* regulating the region *k*. We, therefore, generate a discrete posterior probability distribution of all TF modules for each region. The final number of regulatory modules for each region corresponds to the number of modes in the posterior module distribution. The accumulated samples for each module across all regions proportionally reflect the abundance of this module in the given context. More details about the sampling procedure are given in **S3 Text**.

### ChIP-GSM: TF module-based active regulatory region prediction

The TF module-region posterior regulation matrix $\hat{C}$ is further used to predict active regulatory regions under the assumption that a region bound by multiple TFs is more likely an active regulatory element. By combining ChIP-GSM inferred TF module-region posterior probabilities with experimentally measurements of regulatory region activities in the same context (i.e., breast cancer MCF-7 cells), we train a binomial model using elastic net logistic regression [42] and predict the active/non-active regulatory elements bound by TF modules. Elastic net regression is a natural fit for this application because the modules (features in this binomial model) are highly correlated (sharing TFs) and tend to be highly grouped. Elastic net regression assigns similar weights to correlated features or removes them altogether by assigning zero weights [17]. Unlike linear regression, elastic net regression extends the method of least squares by adding a regularization (or penalty) that includes the feature weights **β** in the minimization process:

minβ0,β1,…,βM−1K′∑k[zk(β0+∑mc^k,mβm)+log(1+exp(∑mc^k,mβm))]+λppα(β), (15)

$$with\;p_{\alpha }(\beta )=\frac{1-\alpha }{2}\sum_{m}\beta_{m}^{2}+\alpha \sum_{m}\left|\beta_{m}\right|.$$

where *z*_*k*_ is a binary (+/-) label for regions with experimentally determined cell-type specific activity; *K*′ is the total number of labelled regions; *λ*_*p*_ is a non-negative parameter controlling the model complexity; 0≤*α*≤1 controls the relative contributions of ridge regression and LASSO to overall regularization penalty. After the training, we obtain an optimal set of weights including *β*_0_ and *β*_1_,…,*β*_*m*_…,*β*_*M*_ for individual modules.

To examine the prediction performance of ChIP-GSM-learned modules and fairly compare the prediction accuracy to methods featuring TFs and/or HMs bindings as inputs, we divide all regions into training and testing groups. ChIP-GSM parameters like the distribution parameters *γ*_*t*_, *α*_*t*_ and *β*_*t*_, candidate modules **B**, and classifier weights *β*_0_, *β*_1_,…,*β*_*m*_…,*β*_*M*_ are all learned from the training regions only. ChIP-GSM then estimates the probabilities of candidate modules on testing regions and further predicts their regulatory activities. We repeat this experiment 100 times by randomly selecting regions for model training so that any potential bias on region selection can be largely eliminated. The variation of module parameters from the original values (learned from all regions) are assessed in terms of root of mean square error (RMSE) for each parameter estimation under each of the nine selected cell types.

With 80% regions as input, the learned model parameters have very small variations from their original values (RMSE across all TFs/mean across all TFs per parameter < 2%), and on average 86% of candidate modules stay the same (**S4 Table**). In this case, the changes caused by region holdout are similar between promoters and enhancers. The F-measure of ChIP-GSM on testing region activity prediction is higher than that of each comparable method under the same settings (**Fig 4**). In the more challenging experiments where only 50% of regions are used for training, most of the model parameters still hold, with only ~5% variation from the original values. Some less frequent modules are missed during the candidate module searching process so that the similarity candidate modules to the original whole set drops to 70% for promoters and 60% for enhancers (**S4 Table**). As shown in **S2 Fig**, the performance of ChIP-GSM mostly holds even with 50% regions as input because model parameters are accurately estimated, and major modules (regulating regions frequently) are well captured. The F-measure of ChIP-GSM is higher than that of EMERGE, the best performing method among the selected comparable methods.

### Epigenetic markers and chromatin interactions enrichment analysis

H3K27ac and H3K4me1 are enhancer marker proteins while H3K4me3 is often used as a promoter marker protein [26]. We download marker proteins ChIP-seq peaks for MCF7 and K562 cell types from the ENCODE database. For each of the three marker proteins, we label a region ‘+’ if there is at least one ChIP-seq peak within 2k bps from the region center; or we label it as ‘-’. Then, two categories of labeled regions are selected as (H3K27ac ‘+’, H3K4me1 ‘+’, H3K4me3 ‘-’) and (H3K27ac ‘-’, H3K4me1 ‘-’, H3K4me3 ‘+’), representing the valid enhancer and promoters, respectively. We assess the enrichment of labeled regions among the top (e.g., 10%) ChIP-GSM-predicted enhancers or promoters, against the enrichment of labeled regions among the FANTOM5 enhancers or promoters of the same cell type. A hypergeometric *p*-value is calculated as:

p(h≥Htop)=∑HtopHall(Hallh)(Kall−HallKtop−h)/(KallKtop) (16)

where *K*_*all*_ is the number of active enhancers (or promoters) in the FANTOM5 database; *H*_*all*_ is the number of labelled regions among *K*_*all*_; *K*_*top*_ is the number of the top predicted regions by ChIP-GSM; *H*_*top*_ is the number of labelled regions among *K*_*top*_.

Cell type-specific ChIA-PET chromatin interactions are downloaded from the ENCODE database. To eliminate nonsense interactions, we select interactions looping between annotated enhancers and promoters. And for each enhancer or promoter, we count the number of interactions around it. Across all enhancers (or promoters), we assess the correlation between ChIP-GSM predicted enhancer (or promoter) activities and the number of ChIA-PET loops of the same regions by fitting a linear model.

### K562 time-course gene expression analysis

We download a time-course K562 gene expression dataset [19] from the GEO database (GEO accession number: GSE1036). K562 cells (duplicate cultures A & B) were treated with 50 micromolar hemin for 0, 6, 12, 24, 48, 72 hours followed by RNA extraction and gene expression profiling on Affymetrix human U133A arrays. Under each time point, there were two duplicates as A0 and B0 under time point ‘0’ and Ai and Bi under each time point ‘i’. For a pair of TFs, we calculate the Pearson correlation coefficient using their mRNA transcription, assuming associated TFs in the same module are more likely to be active at the same time—though the relationship between the protein activity and mRNA transcription may not be linear. We also select up or down-regulated genes if at any time point *i*, compared to time point ‘0’, the gene expression log2 fold change is larger than 1: |log2(A0)-log2(Ai)|>1, |log2(A0)-log2(Bi)|>1, |log2(B0)-log2(Ai)|>1, and |log2(B0)-log2(Bi)|>1. In total, we collected 1,569 genes and assigned them into eight clusters using hierarchical clustering (**Fig 6D**).

## Supporting information

S1 Text ChIP-GSM workflow. (DOCX)

S2 Text ChIP-GSM inferred TF modules at K562 promoters. (DOCX)

S3 Text Supplementary Methods. (DOCX)

S1 Fig A detailed workflow of the ChIP-GSM approach. (TIF)

S2 Fig Two-fold cross-validation on predicting cell type-specific active regulatory regions. (A) F-measure on active enhancers; (B) F-measure on active promoters.
(TIF)

S3 Fig Scatter plots of ChIP-GSM-predicted regulatory activities and ChIA-PET 3D chromatin interactions in MCF7 and K562 cells, respectively. Rare regions with loop count higher than 30 in MCF7 cells or 50 in K562 cells were plotted at 30 in (A) and (C) or at 50 in (B) and (D).
(TIF)

S4 Fig Top 30 ChIP-GSM inferred TF modules from K562 promoters. (TIF)

S5 Fig 11 TF modules inferred using the Rulefit approach, labeled using the same group color as ChIP-GSM. (TIF)

S6 Fig TF modules inferred by ISA. Module identified by ISA can be roughly clustered into four major groups (recovering the Groups 1, 2, 4 and 6 inferred by ChIP-GSM). However, modules in Groups 5 and 8 are missing in the results of ISA.
(TIF)

S7 Fig TF modules inferred by Plaid. Plaid identified five modules with only high-level large-scale associations captured.
(TIF)

S8 Fig Co-expression of TFs in K562 cells. Gene expression data was downloaded from GEO database with ID: GSE1036. Color bar represents–log10(*p*-value) of Pearson correlation coefficient. Rectangles with different colors represent the ChIP-GSM identified module groups.
(TIF)

S9 Fig Target genes regulated by ChIP-GSM inferred modules at K562 promoters. (TIF)

S10 Fig Histograms of ChIP-seq read counts in 500 bps binned promoter regions. Red lines in (A), (C) and (E) represent Power-Law distribution fittings to the TF ChIP-seq read counts. Red lines in (B), (D) and (F) represent Gamma distribution fittings to the input ChIP-seq read counts.
(TIF)

S1 Table ENCODE ChIP-seq profiles used by ChIP-GSM for transcription factor module inference. (XLSX)

S2 Table The similarity of ChIP-GSM-inferred modules between promoter and enhancer regions. (XLSX)

S3 Table ChIP-GSM identified modules. (XLSX)

S4 Table Robustness of ChIP-GSM parameter estimation. (XLSX)

S5 Table Performrance of ChIP-GSM on cell type-specific active regulatory element prediction (F-measure on 20% hold-out regions). (XLSX)

S6 Table Significantly enriched cellular functions in genes regulated by each group of modules in K562 cells. (XLSX)

S7 Table Differentially expressed genes regulated by each group of modules in K562 cells. (XLSX)
