# Supplementary material for: ChIP-GSM: Inferring active transcription factor modules to predict functional regulatory elements
Source: PLoS Comput Biol. 2021 Jul 22;17(7):e1009203. doi: 10.1371/journal.pcbi.1009203 (PMC8330942; doi:10.1371/journal.pcbi.1009203)
Supplement: S1 Text — (DOCX) [file pcbi.1009203.s001.docx]

**ChIP-GSM workflow**

**Step 1**: We use HOMER (v4.9) to process ChIP-seq read count profiles (BAM files; aligned to human reference genome hg19) into read tag files where 5’ start locations as well as directions of read tags in each chromosome are stored in an individual file.

**Step 2**: We partition the input genomic segments into 500 bps bins (regions less than 500 bps will be round up to 500 bps around the original region center) and count reads uniquely aligned to each segment.

**Step 3**: We normalize the total number of reads in each ChIP-seq profile to 10^7^. This step is necessary and important to eliminate any bias caused by the sequencing depth when we pool multiple ChIP-seq profiles for a joint analysis.

**Step 4**: ChIP-GSM can either take an input of pre-generated TF modules or automatically identify a set of candidate modules based on the given ChIP-seq data.

**Step 5**: We initiate the model by assigning possible modules to each region. Then, based on the read counts, we determine regions potentially with or without bindings of each TF. For example, a region with more than 10 reads or 2 folds to the control ChIP-seq profile is likely a TF-bound region (a foreground region); otherwise, it is a background region. We roughly estimate the total number of reads sequenced from TF-bound or background regions. After that, we process these two types of regions separately as we assume their read counts follow distinct distributions.

**Step 6**: We calculate a weight for each TF-bound region based on its read count, by assuming a Power-Law distribution. Here, the Power-Law distribution parameters are TF-specific, which can be obtained by fitting the read count distribution in the TF ChIP-seq profile. We assign reads (the total number is determined in **Step 5)** one by one to all TF-bound regions according to their weights.

**Step 7**: For the same TF but its background regions, we calculate a weight for each based on its read count and a Gamma distribution assumption. Parameters of this Gamma distribution are obtained by fitting read counts in the matched control ChIP-seq profile. We assign the remaining read tags in current TF ChIP-seq profile one by one to background regions according to their weights.

**Step 8**: Specially for promoters, for each TF, we estimate the mean parameter for the Exponential distribution modelling the relative distance of foreground regions to the nearest transcription starting site (TSS), using their genomic locations. **Steps 5 ~ 8** are repeated for each TF.

**Step 9:** We estimate the variance of the residuals between observed and assigned read counts across all segments of all TFs.

**Step 10:** For each region, we calculate a conditional probability for each candidate module given the assigned read counts and probabilistically select a module according to their conditional probability distribution. This current region is classified as a bound region for TFs within the sampled module or otherwise a background region. Results of **Step 10** are recorded and then brought back to **Step 5** to initiate the next round of sampling.

We run the sampling process until the sampler appears to converge on the equilibrium distribution and then start accumulating samples on module-region units. After collecting enough samples, the sampling frequency of each module-region unit denotes the posterior probability for binding occurrence.
