# Supplementary material for: ChIP-GSM: Inferring active transcription factor modules to predict functional regulatory elements
Source: PLoS Comput Biol. 2021 Jul 22;17(7):e1009203. doi: 10.1371/journal.pcbi.1009203 (PMC8330942; doi:10.1371/journal.pcbi.1009203)
Supplement: S2 Text — (DOCX) [file pcbi.1009203.s002.docx]

**ChIP-GSM inferred TF modules at K562 promoters**

In Group 1, the similarity for TFs across modules is very high (red block in **S4 Fig**). Two associations, RBBP5-PHF8-SAP30-HDAC1 and EGR1-ZBTB7A, were previously validated in K562 cells [[1](#_ENREF_1),[2](#_ENREF_2)]. A novel association PHF8–ZBTB7A is predicted in two modules; the *p*-value of their mRNA expression correlation is 3.75e-2.

In Group 2, all TFs (i.e., CBX2, CBX8, RNF2, EZH2 and SUZ12) are Polycomb-group proteins whose associations have been established under stem cells and also verified to be present in K562 cells [[3](#_ENREF_3)]. Their mRNA expression are quite diverse and we did not found significant correlation.

In Group 3, pairwise correlation is highlighted but there is no significant triple wise correlation (**S8 Fig**). The module GATA1-GATA2-TAL1 has been previously observed in [[2](#_ENREF_2)] based on whole genome study. With existing evidence in support of the associations of GATA3-GATA2 and GATA3-TEAD4 [[4](#_ENREF_4), [5](#_ENREF_5)], a high-order association of GATA2-GATA3-TEAD4 is expected. However, due to the lack of GATA3 ChIP-seq data, only GATA2-TEAD4 is identified; the *p*-value of mRNA expression correlation is 3.29e-2. GATA2-ZBTB33 was previously confirmed by an independent study by checking co-binding between GATA2 and ZBTB33 in K562 cells [[6](#_ENREF_6)].

In Group 4, TF combinations are more diverse, with weak evidence of mRNA expression correlation. The co-binding of RBBP5 and CHD1 has been observed at active promoters with enrichment of H3K4me3 [[7](#_ENREF_7), [8](#_ENREF_8)]. SIRT6 is also highly enriched at active promoters as well and its association with CHD1 is related to productive initiation [[3](#_ENREF_3)].

Group 5 is a large module group where two high-order associations, SP2-NFYA-NFYB and FOSL1-FOS-JUND-CEBPB, are validated to exist in K562 cells [[9](#_ENREF_9)]. The correlation of mRNA expression for Group 5 TFs is very strong (purple block in **S8 Fig**). Here, a novel association, FOSL1-NFYA-NFYB, is predicted by ChIP-GSM.

In Group 6, RAD21, SMC3 and CTCF are members of the cohesin complex [[10](#_ENREF_10),[11](#_ENREF_11)], with highly significant mRNA expression correlation. CTCFL is a paralog of CTCF. Its interaction with RAD21 or SMC3 is also supported by their binding signal enrichment at the same regions.

Group 7 only has three TFs but the association between BACH1 and MAF protein complex including MAFF and MAFK is very strong, especially with MAFK to form a heterodimer [[12](#_ENREF_12)]. In **S8** **Fig** (green block), BACH1 has a strong correlation with either MAFF or MAFK.

In Group 8, E2F6 is recruited by MAX to gene promoters via E boxes (CACGTG) via protein-protein interaction [[13](#_ENREF_13)]. MAX, USF1, and BHLHE40 (BHLHB2) are E-box binding factors so that two associations of them including MAX-USF1 and BHLHE40-USF1 are identified by the ChIP-GSM approach. The mRNA expression correlation between E2F6 and MAX is significant. Since MAX and BHLHE40 are indirectly connected with USF1 (the expression profile of which is not available in our study), the correlation of MAX and BHLHE40 is weaker but still significant.

# References

1. Giannopoulou EG, Elemento O. Inferring chromatin-bound protein complexes from genome-wide binding assays. Genome research. 2013;23(8):1295-306. doi: 10.1101/gr.149419.112. PubMed PMID: 23554462; PubMed Central PMCID: PMC3730103.

2. Gerstein MB, Kundaje A, Hariharan M, Landt SG, Yan KK, Cheng C, et al. Architecture of the human regulatory network derived from ENCODE data. Nature. 2012;489(7414):91-100. doi: 10.1038/nature11245. PubMed PMID: 22955619; PubMed Central PMCID: PMC4154057.

3. Ram O, Goren A, Amit I, Shoresh N, Yosef N, Ernst J, et al. Combinatorial patterning of chromatin regulators uncovered by genome-wide location analysis in human cells. Cell. 2011;147(7):1628-39. doi: 10.1016/j.cell.2011.09.057. PubMed PMID: 22196736; PubMed Central PMCID: PMC3312319.

4. Ralston A, Cox BJ, Nishioka N, Sasaki H, Chea E, Rugg-Gunn P, et al. Gata3 regulates trophoblast development downstream of Tead4 and in parallel to Cdx2. Development. 2010;137(3):395-403. doi: 10.1242/dev.038828. PubMed PMID: 20081188.

5. Home P, Saha B, Ray S, Dutta D, Gunewardena S, Yoo B, et al. Altered subcellular localization of transcription factor TEAD4 regulates first mammalian cell lineage commitment. Proceedings of the National Academy of Sciences of the United States of America. 2012;109(19):7362-7. doi: 10.1073/pnas.1201595109. PubMed PMID: 22529382; PubMed Central PMCID: PMC3358889.

6. Blattler A, Yao L, Wang Y, Ye Z, Jin VX, Farnham PJ. ZBTB33 binds unmethylated regions of the genome associated with actively expressed genes. Epigenetics & chromatin. 2013;6(1):13. doi: 10.1186/1756-8935-6-13. PubMed PMID: 23693142; PubMed Central PMCID: PMC3663758.

7. Smith E, Shilatifard A. The chromatin signaling pathway: diverse mechanisms of recruitment of histone-modifying enzymes and varied biological outcomes. Molecular cell. 2010;40(5):689-701. doi: 10.1016/j.molcel.2010.11.031. PubMed PMID: 21145479; PubMed Central PMCID: PMC3037032.

8. Sims RJ, 3rd, Chen CF, Santos-Rosa H, Kouzarides T, Patel SS, Reinberg D. Human but not yeast CHD1 binds directly and selectively to histone H3 methylated at lysine 4 via its tandem chromodomains. The Journal of biological chemistry. 2005;280(51):41789-92. doi: 10.1074/jbc.C500395200. PubMed PMID: 16263726; PubMed Central PMCID: PMC1421377.

9. Ernst J, Kellis M. Interplay between chromatin state, regulator binding, and regulatory motifs in six human cell types. Genome research. 2013;23(7):1142-54. doi: DOI 10.1101/gr.144840.112. PubMed PMID: WOS:000321119900011.

10. Wendt KS, Peters JM. How cohesin and CTCF cooperate in regulating gene expression. Chromosome Res. 2009;17(2):201-14. doi: DOI 10.1007/s10577-008-9017-7. PubMed PMID: WOS:000264515300007.

11. Xie D, Boyle AP, Wu L, Zhai J, Kawli T, Snyder M. Dynamic trans-acting factor colocalization in human cells. Cell. 2013;155(3):713-24. doi: 10.1016/j.cell.2013.09.043. PubMed PMID: 24243024; PubMed Central PMCID: PMC4079469.

12. Okita Y, Kamoshida A, Suzuki H, Itoh K, Motohashi H, Igarashi K, et al. Transforming growth factor-beta induces transcription factors MafK and Bach1 to suppress expression of the heme oxygenase-1 gene. The Journal of biological chemistry. 2013;288(28):20658-67. doi: 10.1074/jbc.M113.450478. PubMed PMID: 23737527; PubMed Central PMCID: PMC3711329.

13. Ogawa H, Ishiguro K, Gaubatz S, Livingston DM, Nakatani Y. A complex with chromatin modifiers that occupies E2F-and Myc-responsive genes in G(0) cells. Science. 2002;296(5570):1132-6. doi: DOI 10.1126/science.1069861. PubMed PMID: WOS:000175565000058.
