# Supplementary material for: ChIP-GSM: Inferring active transcription factor modules to predict functional regulatory elements
Source: PLoS Comput Biol. 2021 Jul 22;17(7):e1009203. doi: 10.1371/journal.pcbi.1009203 (PMC8330942; doi:10.1371/journal.pcbi.1009203)
Supplement: S3 Text — (DOCX) [file pcbi.1009203.s003.docx]

# Supplementary Methods

**Probabilistic distribution hypothesizes**

$X_{k,t}$ (read count at foreground region with $b\left( c_{k},t \right)=1$) follows a Power-Law distribution:

${P(X}_{k,t}) \sim\frac{\gamma_{t}-1}{X_{min}}{(\frac{X_{k,t}}{X_{min}})}^{-\gamma_{t}}$. (S-1)

$I_{k,t}$ (read count at background region with $b\left( c_{k},t \right)=0$) follows a Gamma distribution:

${P(I}_{k,t}) \sim{I_{k,t}}^{\alpha_{t}-1}exp(-\beta_{t}I_{k,t})$. (S-2)

$N_{k,t}$ (read count noise at each region) follows a Gaussian distribution:

${P(N}_{k,t}) \sim\frac{1}{\sigma_{N}}exp(-\frac{{N_{k,t}}^{2}}{2\sigma_{N}^{2}})$. (S-3)

$\sigma_{N}^{2}$ (noise variance at all regions) follows an Inverse Gamma distribution:

$P\left( \sigma_{N}^{2} \right) \sim\left( \sigma_{N}^{2} \right)^{\alpha_{t}-1}\exp\left( -\frac{\beta_{t}}{\sigma_{N}^{2}} \right)$. (S-4)

$d_{k}$ (relative distance of each region to the nearest TSS) follows Exponential distribution for $b\left( c_{k},t \right)=1$ or Uniform distribution for $b\left( c_{k},t \right)=0$:

$\left\{ \begin{aligned} P\left( d_{k} | b\left( c_{k},t \right)=1 \right) \sim\lambda_{t}exp(-\lambda_{t}|d_{k}|) \\ P\left( d_{k,t} | b\left( c_{k},t \right)=0 \right) \sim{\Delta d/d}_{p} \end{aligned} \right.$, (S-5)

**Gibbs sampling procedure**

**(1) Estimate the total number of tags**

Based on initial selection or previous round of sampling, we have a module-region regulation matrix $\mathbf{C}$ as well as the sampled binding state $b\left( c_{k},t \right)$ between every TF $t$ and every region $k$. For any region with state $b\left( c_{k},t \right)=1$, we amplify its weight $p_{k,t}$ by $F$ times. Then we roughly estimate the total number of reads assigned to foreground regions as follows:

$R_{X,t}=\frac{R_{t}F\sum_{k} b\left( c_{k},t \right)p_{k,t}}{F\sum_{k} b\left( c_{k},t \right)p_{k,t}+\sum_{k} \left( 1-b\left( c_{k},t \right) \right)p_{k,t}}$, (S-6)

where $R_{t}$ is the total number of read tags of TF $t$. The total number of reads $R_{I,t}$ assigned to background regions can be calculated as $R_{t}-R_{X,t}$.

**(2) Sampling read counts for foreground regions**

We sample a read count ${X'}_{k,t}$ for a binding event $b\left( c_{k},t \right)=1$ according to the posterior probability density function as follows:

$P(X|b\left( c_{k},t \right)=1)\propto\frac{1}{\sigma_{N}}exp(-\frac{{{(Y}_{k,t}-X)}^{2}}{2\sigma_{N}^{2}})\frac{\gamma_{t}-1}{X_{min}}{(\frac{X}{X_{min}})}^{-\gamma_{t}}\lambda_{t}exp(-\lambda_{t}|d_{k}|)$. (S-7)

Then, the weight of current region, $p_{k,t}$, is calculated based on the sampled ${X'}_{k,t}$ and the above discrete probability density function. Based on our hypothesis of PowerLaw distribution for the read count of $b\left( c_{k},t \right)=1$, we first assign $X_{min}$ reads to each of such regions, then probabilistically assign the remaining $R_{X,t}-X_{min}\sum_{k} b(c_{k},t)$ reads to all such regions based on their weights and finally obtain the estimated read count $X_{k,t}$ for each foreground region.

**(3) Sampling read counts for background regions**

We sample a read count ${I'}_{k,t}$ for a non-binding event $b\left( c_{k},t \right)=0$ according to the posterior probability density function as follows:

$P(I|b\left( c_{k},t \right)=0)\propto\frac{1}{\sigma_{N}}\exp\left( -\frac{{{(Y}_{k,t}-I)}^{2}}{2\sigma_{N}^{2}} \right)I^{\alpha_{t}-1}\exp\left( -\beta_{t}I \right)\frac{\Delta d}{d_{p}}$. (S-8)

Then, the weight of current region, $p_{k,t}$, is calculated based on the sampled ${I'}_{k,t}$ and the above discrete probability density function. We probabilistically assign $R_{t}-R_{X,t}$ reads to all such regions based on their weights and finally obtain the estimated read count $I_{k,t}$ for each background region.

**(4) Sampling variance of read count residuals**

After estimating read counts (${Y'}_{k,t}$ = $b\left( c_{k},t \right){X'}_{k,t}+\left( 1-b\left( c_{k},t \right) \right){I'}_{k,t}$) for every region, across all regions, we sample $\sigma_{N}^{2}$, the variance of residuals between observed read counts $\mathbf{Y}$ and estimated read counts $\mathbf{Y}'$, according to its posterior probability distribution as follows:

$P\left( \sigma_{N}^{2}|\mathbf{Y},\mathbf{Y}' \right) \sim\prod_{k,t} \frac{1}{\sigma_{N}}exp(-\frac{{(Y_{k,t}-{Y^{'}}_{k,t})}^{2}}{2\sigma_{N}^{2}})\left( \sigma_{N}^{2} \right)^{\alpha_{t}-1}\exp\left( -\frac{\beta_{t}}{\sigma_{N}^{2}} \right)$. (S-9)

Eq. (S-9) is still an Inverse-gamma distribution, so we are able to draw samples of $\sigma_{N}^{2}$ directly using updated distribution parameters as $\alpha_{N}+\frac{KT}{2}$ and $\beta_{N}+\sum_{k,t} {({Y_{k,t}-Y'}_{k,t})}^{2}/2$.

**(5) Sampling TF modules**

For each region, the posterior probability for a candidate module is estimated as follows:

$P\left( c_{k}=m | \mathbf{Y,X,I,D, B} \right)=\frac{\prod_{t} P(Y_{k,t}|{Y^{'}}_{k,t})P({Y^{'}}_{k,t})P(d_{k}|b(m,t))}{\sum_{j} \prod_{t} P(Y_{k,t}|{Y^{'}}_{k,t})P({Y^{'}}_{k,t})P(d_{k}|b(j,t))}$. (S-10)

We calculate the posterior probability of every candidate module for the same region and probabilistically sample a module as the inferred one. Then, we update the binding state of every region to initiate the next round of sampling by repeating Steps (1) to (5). After accumulating enough samples, the final number of regulatory modules for each region corresponds to the number of modes in the posterior sample distribution.

**Elastic-net logistic regression model parameters**

The MATLAB version of Elastic Net Logistic Regression package can be downloaded from <https://web.stanford.edu/~hastie/glmnet_matlab/>.

Parameter settings are listed as follows:

options.alpha = 0.1;

options.nlambda = 100;

options.standardize = true;

options.intr = true;

options.thresh = 1e-7;

options.cl = [-Inf;Inf];

options.maxit = 1e+5;

options.ltype = 'Newton';

options.standardize_resp = false;

options.mtype = 'ungrouped';

family = 'binomial';
