# Supplementary figures and images for: ChIP-GSM: Inferring active transcription factor modules to predict functional regulatory elements
Source: PLoS Comput Biol. 2021 Jul 22;17(7):e1009203. doi: 10.1371/journal.pcbi.1009203 (PMC8330942; doi:10.1371/journal.pcbi.1009203)

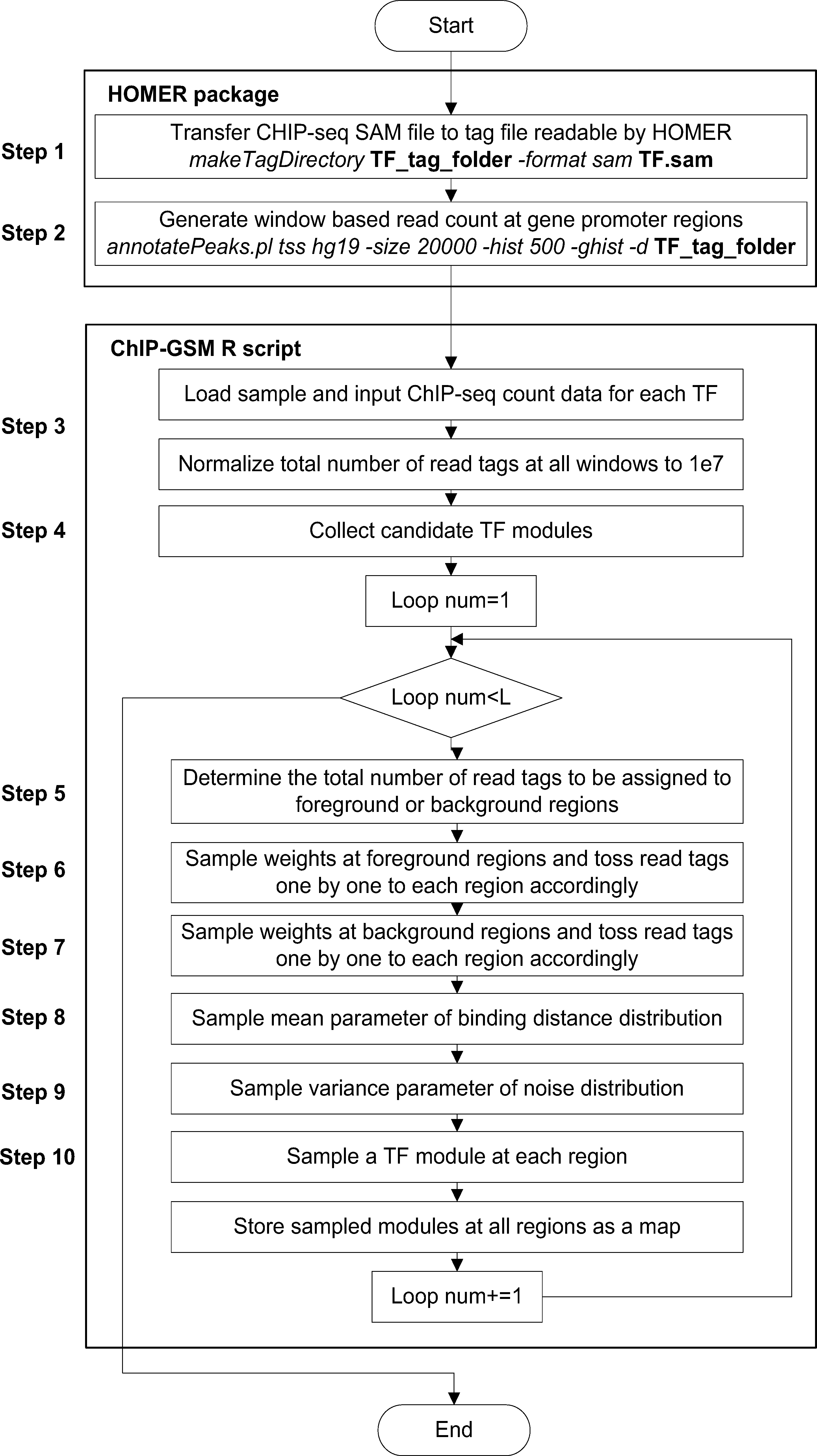

Supplement: S1 Fig — (TIF) [file pcbi.1009203.s004.tif]

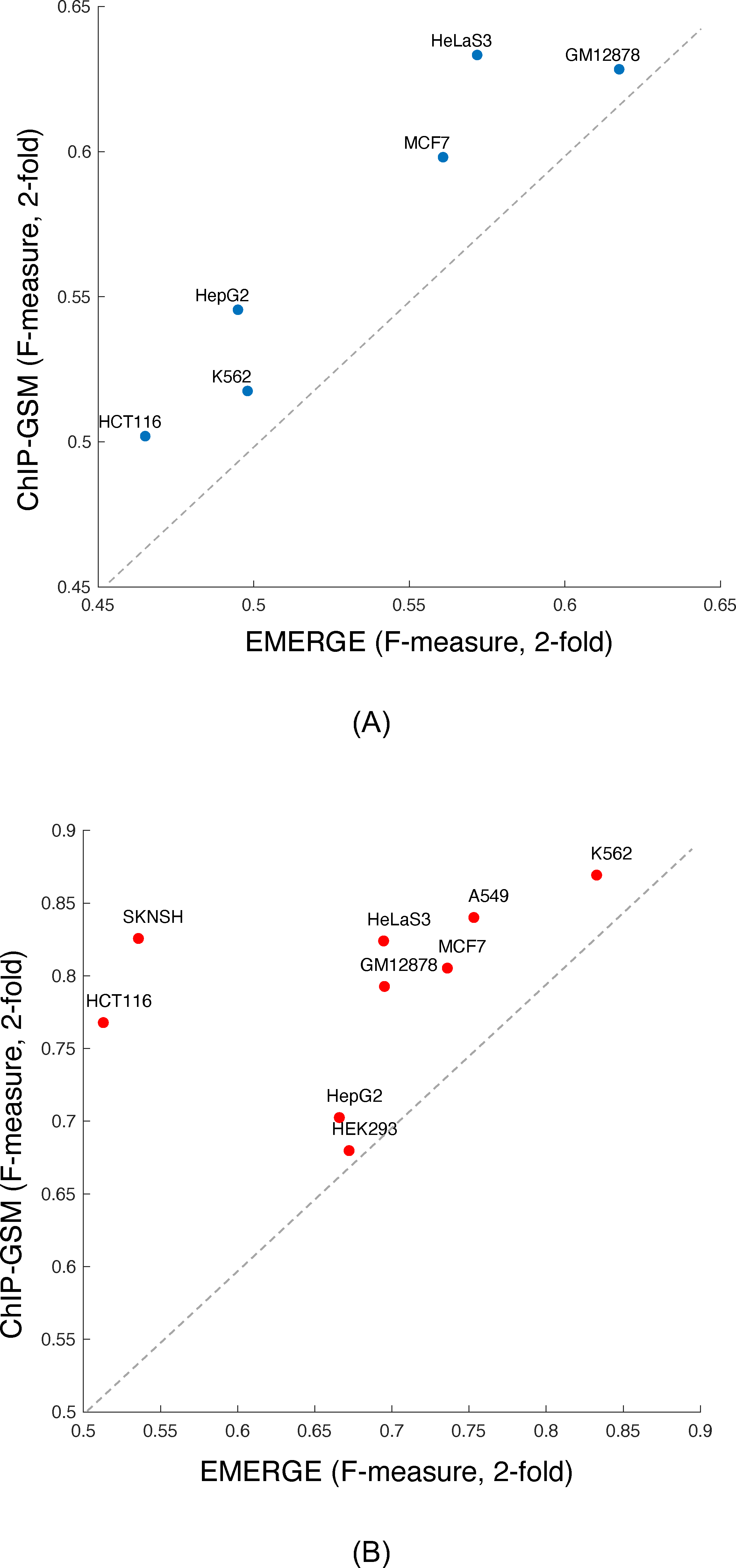

Supplement: S2 Fig — (A) F-measure on active enhancers; (B) F-measure on active promoters. (TIF) [file pcbi.1009203.s005.tif]

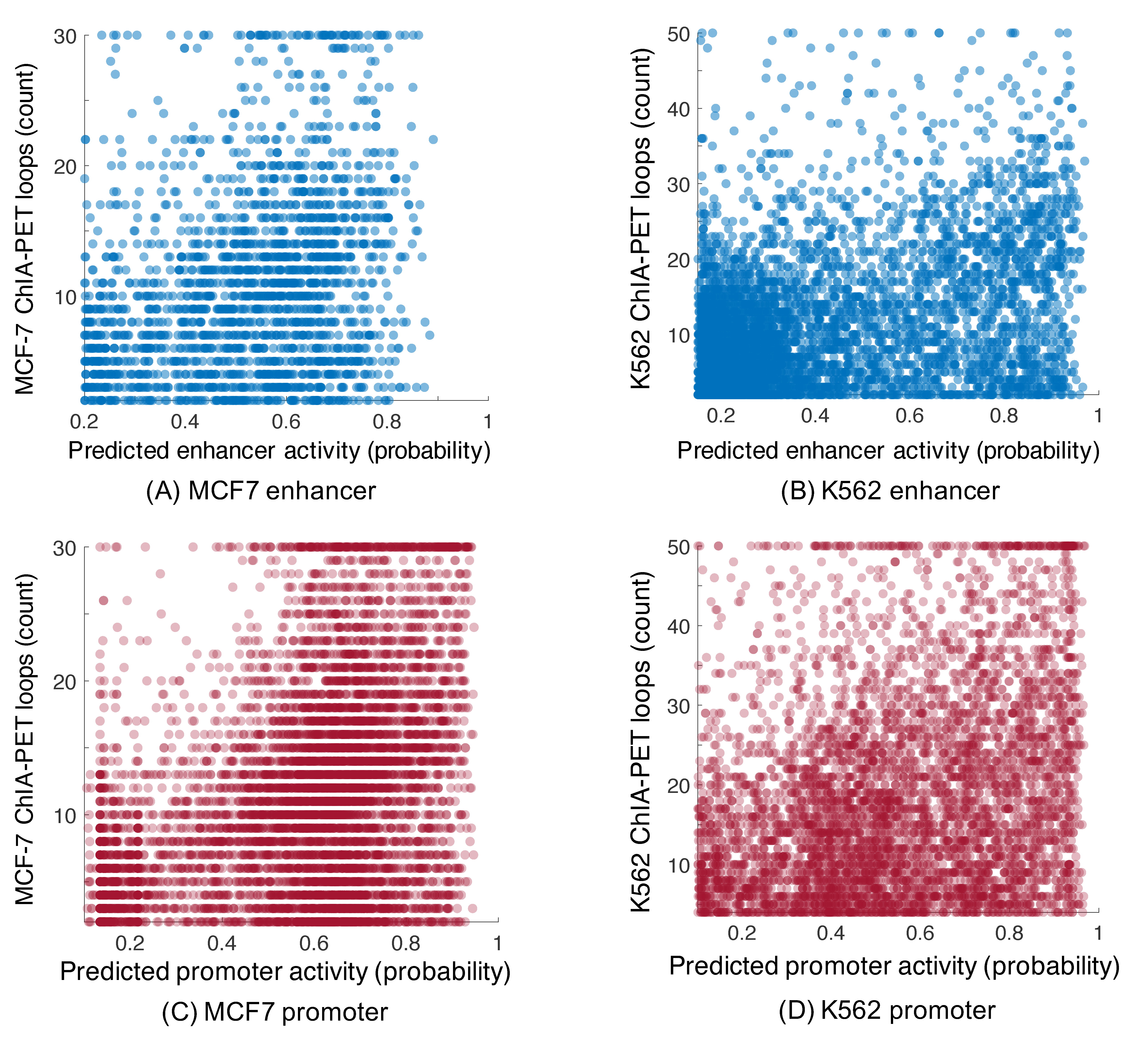

Supplement: S3 Fig — Rare regions with loop count higher than 30 in MCF7 cells or 50 in K562 cells were plotted at 30 in (A) and (C) or at 50 in (B) and (D). (TIF) [file pcbi.1009203.s006.tif]

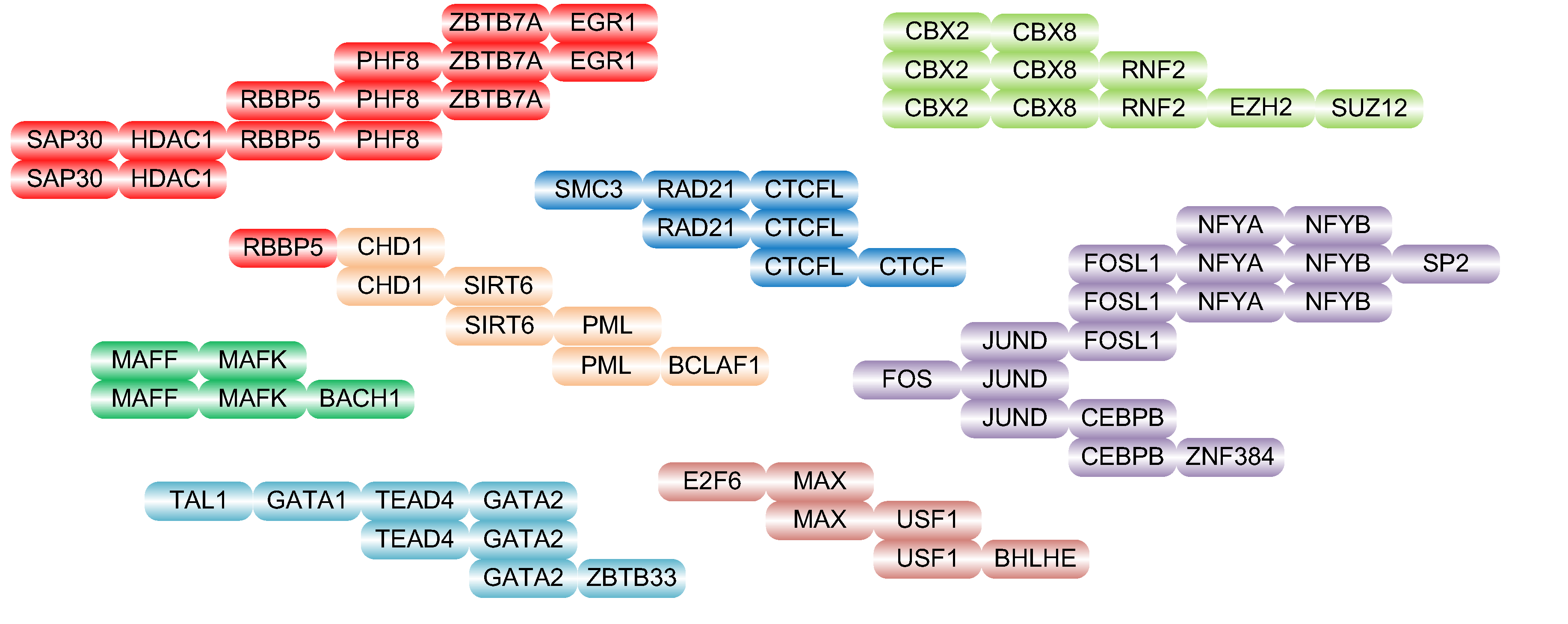

Supplement: S4 Fig — (TIF) [file pcbi.1009203.s007.tif]

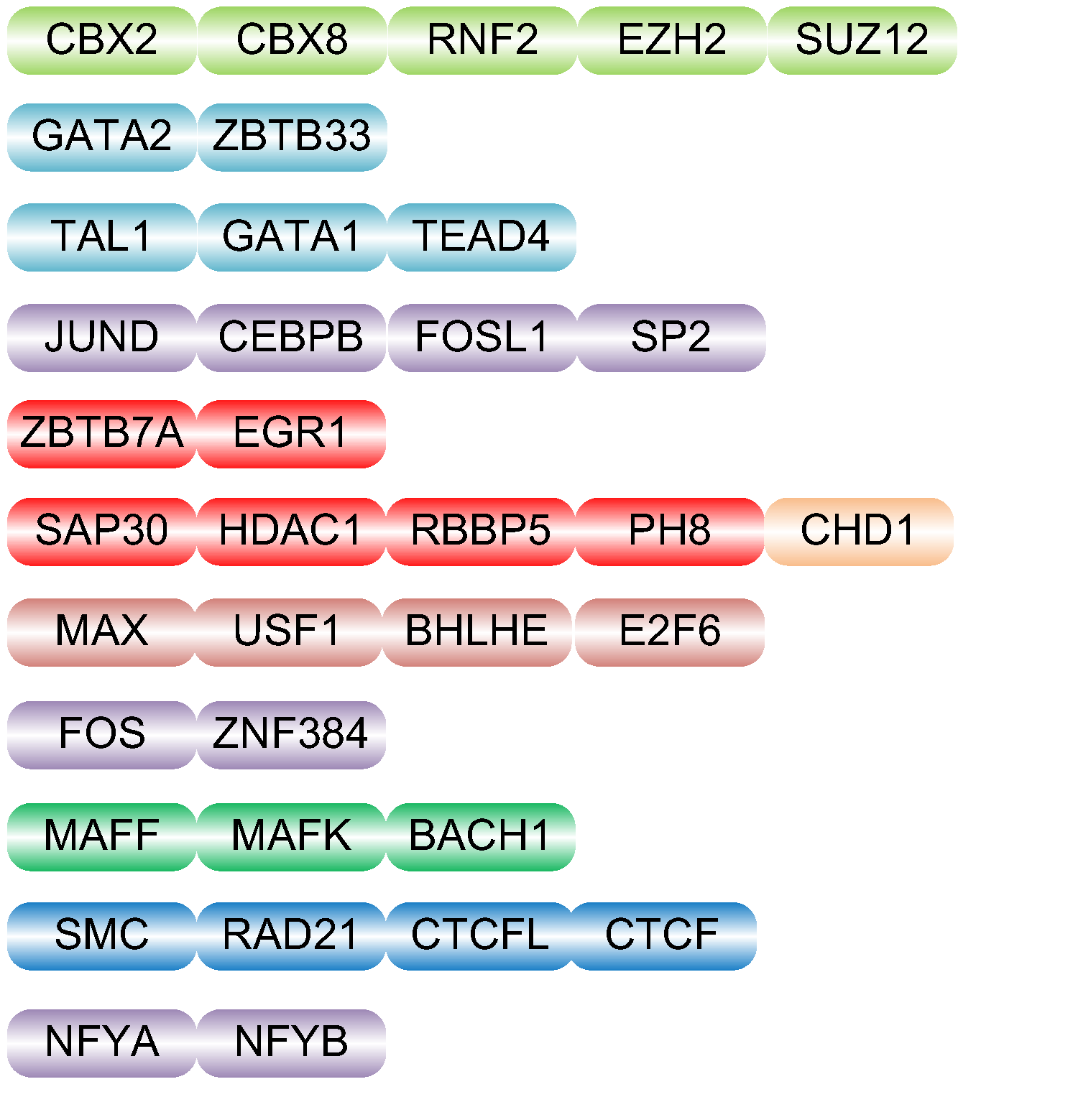

Supplement: S5 Fig — (TIF) [file pcbi.1009203.s008.tif]

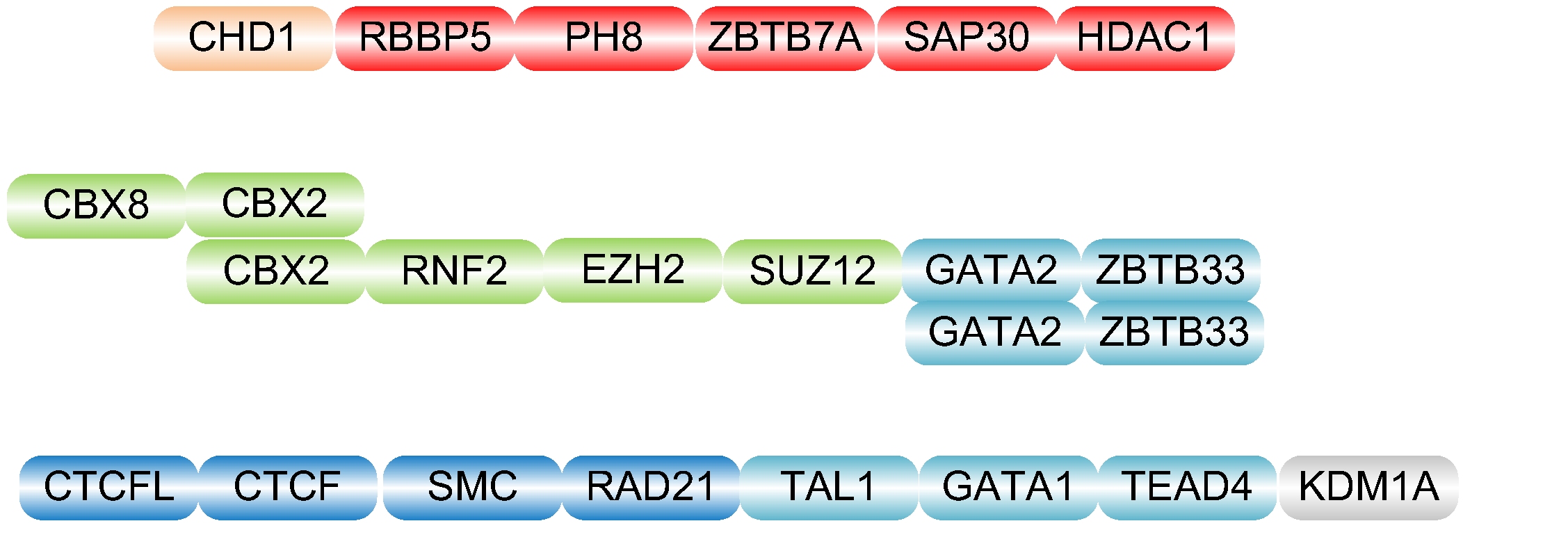

Supplement: S7 Fig — Plaid identified five modules with only high-level large-scale associations captured. (TIF) [file pcbi.1009203.s010.tif]

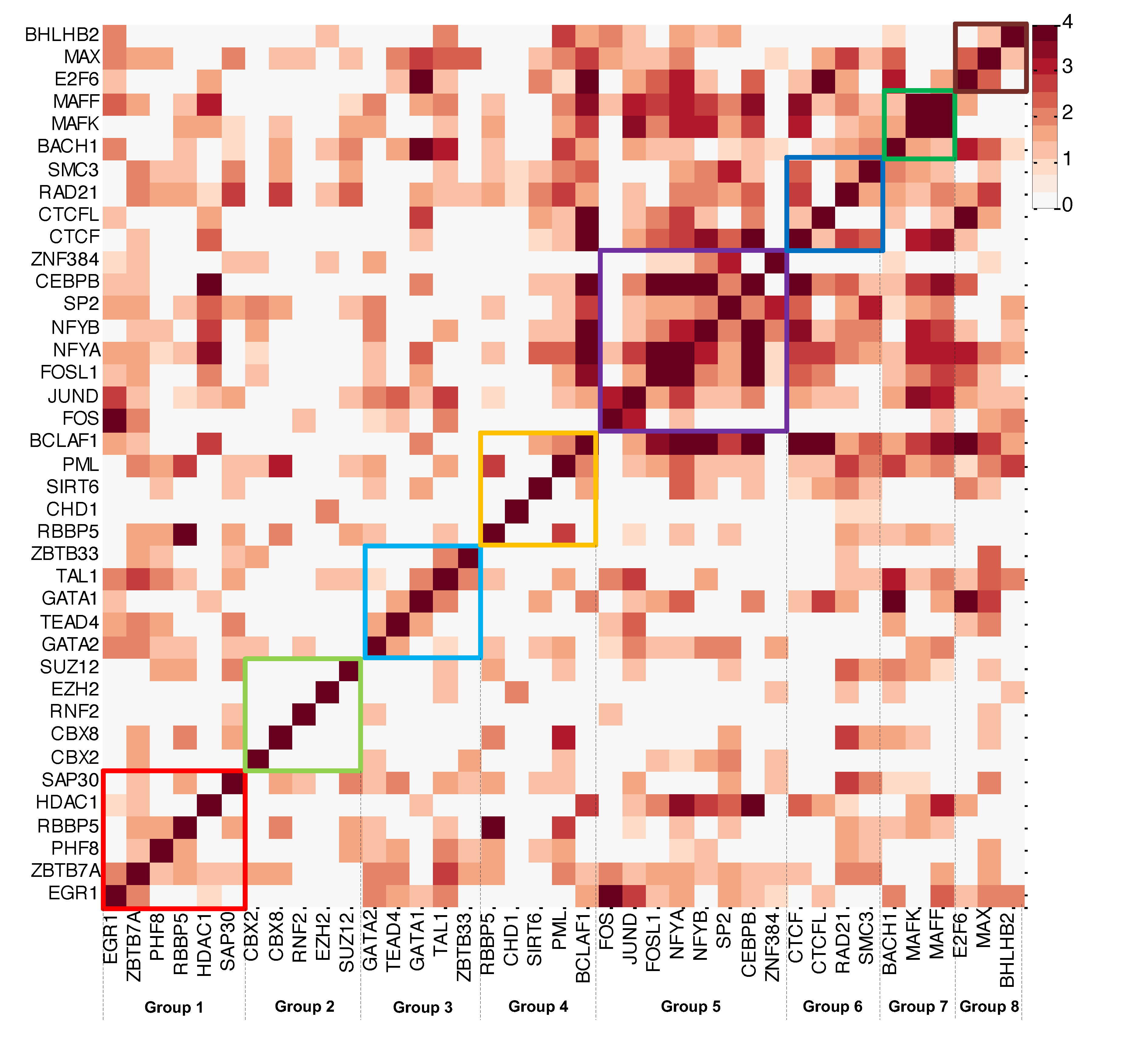

Supplement: S8 Fig — Gene expression data was downloaded from GEO database with ID: GSE1036. Color bar represents–log10(p-value) of Pearson correlation coefficient. Rectangles with different colors represent the ChIP-GSM identified module groups. (TIF) [file pcbi.1009203.s011.tif]

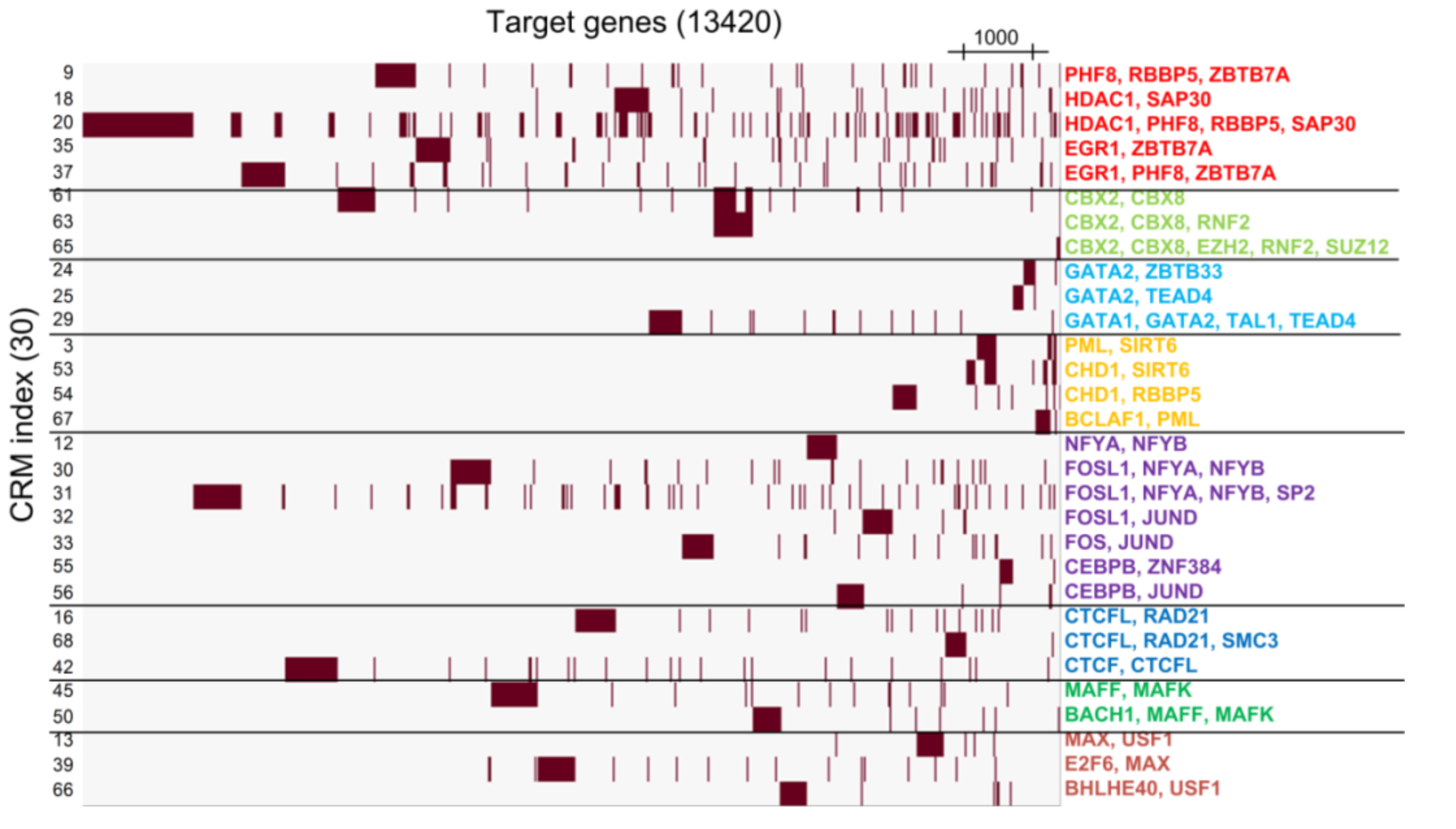

Supplement: S9 Fig — (TIF) [file pcbi.1009203.s012.tif]

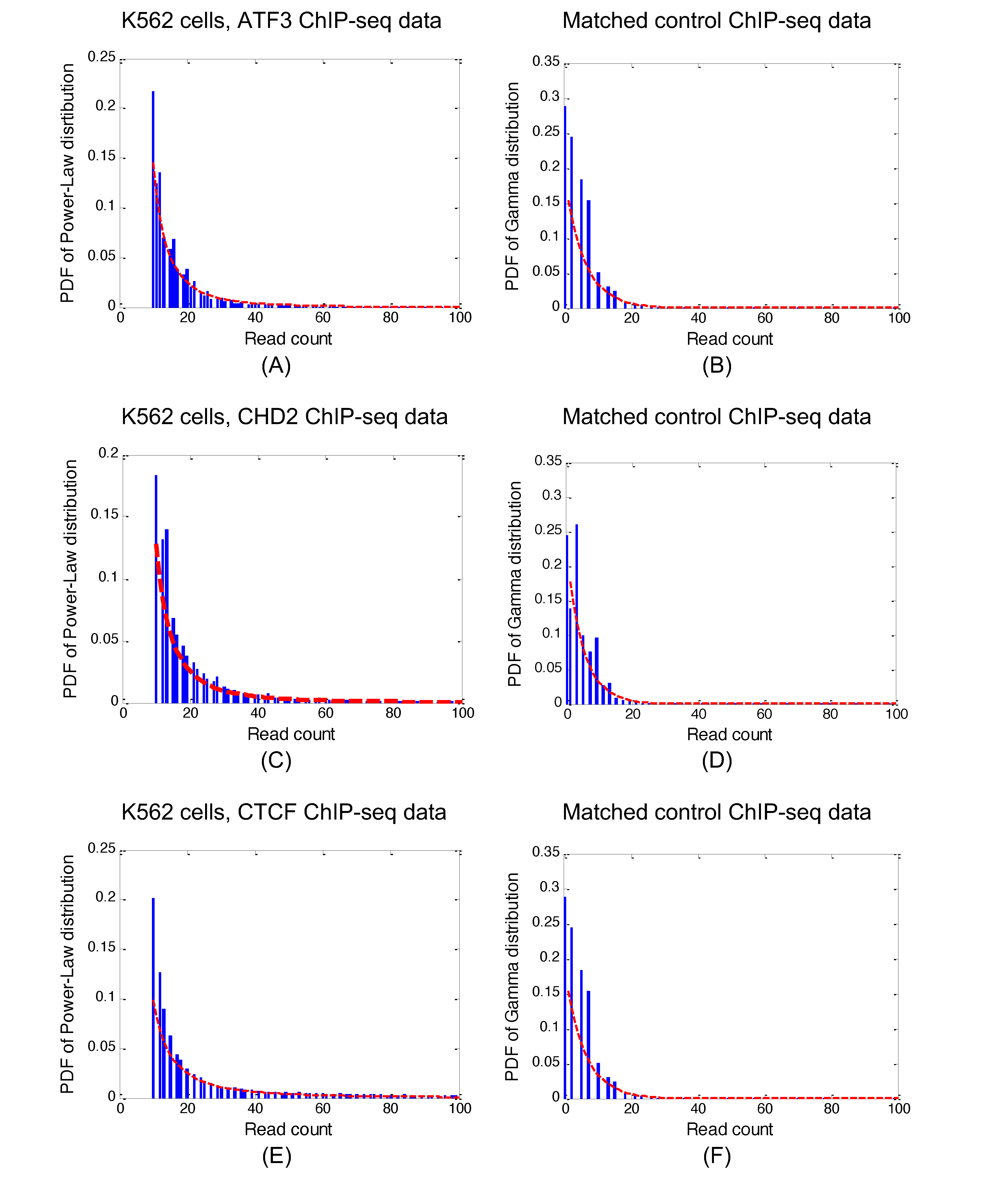

Supplement: S10 Fig — Red lines in (A), (C) and (E) represent Power-Law distribution fittings to the TF ChIP-seq read counts. Red lines in (B), (D) and (F) represent Gamma distribution fittings to the input ChIP-seq read counts. (TIF) [file pcbi.1009203.s013.tif]
